# Modular organization of enhancer network provides transcriptional robustness in mammalian development

**DOI:** 10.1093/nar/gkae1323

**Published:** 2025-01-16

**Authors:** Hongli Lin, Xinyun Ye, Wenyan Chen, Danni Hong, Lifang Liu, Feng Chen, Ning Sun, Keying Ye, Jizhou Hong, Yalin Zhang, Falong Lu, Lei Li, Jialiang Huang

**Affiliations:** State Key Laboratory of Cellular Stress Biology, Xiang’an Hospital, School of Life Sciences, Faculty of Medicine and Life Sciences, Xiamen University, No. 4221, Xiang’an South Road, Xiamen, Fujian 361102, China; State Key Laboratory of Cellular Stress Biology, Xiang’an Hospital, School of Life Sciences, Faculty of Medicine and Life Sciences, Xiamen University, No. 4221, Xiang’an South Road, Xiamen, Fujian 361102, China; Institute of Systems and Physical Biology, Shenzhen Bay Laboratory, Guangqiao Road, Shenzhen 518055, China; State Key Laboratory of Cellular Stress Biology, Xiang’an Hospital, School of Life Sciences, Faculty of Medicine and Life Sciences, Xiamen University, No. 4221, Xiang’an South Road, Xiamen, Fujian 361102, China; State Key Laboratory of Cellular Stress Biology, Xiang’an Hospital, School of Life Sciences, Faculty of Medicine and Life Sciences, Xiamen University, No. 4221, Xiang’an South Road, Xiamen, Fujian 361102, China; Chengdu Wiser Matrix Technology Co. Ltd, No. 399, Fucheng Road, Chengdu, Sichuan 614001, China; Chengdu Wiser Matrix Technology Co. Ltd, No. 399, Fucheng Road, Chengdu, Sichuan 614001, China; State Key Laboratory of Cellular Stress Biology, Xiang’an Hospital, School of Life Sciences, Faculty of Medicine and Life Sciences, Xiamen University, No. 4221, Xiang’an South Road, Xiamen, Fujian 361102, China; State Key Laboratory of Cellular Stress Biology, Xiang’an Hospital, School of Life Sciences, Faculty of Medicine and Life Sciences, Xiamen University, No. 4221, Xiang’an South Road, Xiamen, Fujian 361102, China; State Key Laboratory of Cellular Stress Biology, Xiang’an Hospital, School of Life Sciences, Faculty of Medicine and Life Sciences, Xiamen University, No. 4221, Xiang’an South Road, Xiamen, Fujian 361102, China; State Key Laboratory of Molecular Developmental Biology, Institute of Genetics and Developmental Biology, Chinese Academy of Sciences, No. 2, Beichen West Road, Beijing 100101, China; College of Advanced Agricultural Sciences, University of Chinese Academy of Sciences, No. 1, Yanqihu East Road, Beijing 101408, China; Institute of Systems and Physical Biology, Shenzhen Bay Laboratory, Guangqiao Road, Shenzhen 518055, China; State Key Laboratory of Cellular Stress Biology, Xiang’an Hospital, School of Life Sciences, Faculty of Medicine and Life Sciences, Xiamen University, No. 4221, Xiang’an South Road, Xiamen, Fujian 361102, China; National Institute for Data Science in Health and Medicine, Xiamen University, No. 4221, Xiang’an South Road, Xiamen, Fujian 361102, China

## Abstract

Enhancer clusters, pivotal in mammalian development and diseases, can organize as enhancer networks to control cell identity and disease genes; however, the underlying mechanism remains largely unexplored. Here, we introduce eNet 2.0, a comprehensive tool for enhancer networks analysis during development and diseases based on single-cell chromatin accessibility data. eNet 2.0 extends our previous work eNet 1.0 by adding network topology, comparison and dynamics analyses to its network construction function. We reveal modularly organized enhancer networks, where inter-module interactions synergistically affect gene expression. Moreover, network alterations correlate with abnormal and dynamic gene expression in disease and development. eNet 2.0 is robust across diverse datasets. To facilitate application, we introduce eNetDB (https://enetdb.huanglabxmu.com), an enhancer network database leveraging extensive scATAC-seq (single-cell assay for transposase-accessible chromatin sequencing) datasets from human and mouse tissues. Together, our work provides a powerful computational tool and reveals that modularly organized enhancer networks contribute to gene expression robustness in mammalian development and diseases.

## Graphical Abstract

**Graphical Abstract:**
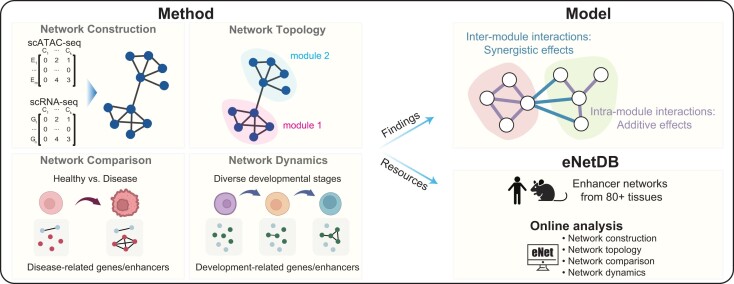

## Introduction

Enhancers are non-coding DNA *cis-*regulatory elements that serve a crucial role in enhancing the frequency of gene transcription (1,2). Previous studies have revealed that enhancers can form large clusters, also known as shadow enhancers, redundant enhancers, super-enhancers (SEs) or enhancer clusters, to govern the precise spatiotemporal expression of crucial genes that play pivotal roles in development and disease (3–5). However, the underlying mechanisms remain not yet well understood. To this end, various technologies have been employed but showed limitations. For example, the utilization of the CRISPR/Cas9 system for genome editing serves as a valuable tool for investigating enhancer cluster organization (6). However, it has limited throughput and is not suitable for large-scale studies (5,7–14). CRISPR screen overcame the limitation of throughput (15,16), but it is currently constrained to studying well-established enhancer clusters in specific cell lines due to a lack of effective guidance for predicting crucial enhancer clusters in cell fate and disease (17). Furthermore, the implementation of Hi-C technology allows for the generation of genome contact maps through extensive examination of all possible interactions. It has greatly enhanced our comprehension of the intricate three-dimensional organization of the genome (18). Nevertheless, obtaining high-resolution Hi-C data still poses experimental challenges.

The advancement of single-cell technologies, such as single-cell RNA sequencing (scRNA-seq) and single-cell assay for transposase-accessible chromatin sequencing (scATAC-seq), along with their associated computational methods (19–23), has facilitated a deeper comprehension of enhancer–gene regulation. Moreover, these technologies have opened up exciting opportunities to explore the regulatory interplay among constituent enhancers at the single-cell level (24). For example, we recently developed an algorithm eNet to integrate single-cell multi-omics profiles and build enhancer network, which elucidates the enhancer regulatory relationship for each gene (25). We found that the complexity of enhancer networks predicts cell identity and disease genes, validated by *in vivo* CRISPR/Cas9-mediated enhancer knockout experiment (26). However, the topological properties of enhancer networks, as well as their regulatory roles during development and diseases, remain largely unexplored.

In this study, we upgrade eNet algorithm to a comprehensive enhancer network analysis tool, termed eNet 2.0, which extends three modules for network topology, comparison and dynamics analysis. We applied eNet 2.0 on the rich resource of the existing single-cell chromatin accessibility data and built eNetDB, a user-friendly database of enhancer networks across diverse human and mouse tissues. Remarkably, our findings reveal the modular organization of enhancer networks, which contributes to transcriptional robustness in mammalian development. Our network comparison and dynamics analysis can pinpoint disease-specific and stage-specific enhancer networks, showcasing superior performance in identifying disease-related and cell identity genes compared to eNet 1.0 and the existing models. Together, eNet 2.0 provides a valuable tool and resource for researchers to gain deeper insights into the epigenetic mechanisms in gene regulation during disease pathogenesis and development.

## Materials and methods

### Data collection

All scATAC-seq data and matched scRNA-seq data were collected from various sources, including Gene Expression Omnibus (https://www.ncbi.nlm.nih.gov/geo/), the Zenodo data archive (https://zenodo.org/) and Genome Sequence Archive (https://ngdc.cncb.ac.cn/gsa/), as listed in Supplementary Table S1.

### Construction of enhancer networks using eNet analysis

We followed the steps in eNet (25) to construct enhancer networks. First, we prepared the enhancer accessibility and gene expression matrices from scATAC-seq and scRNA-seq profiles (or gene activity as a proxy) as input for eNet. Second, we identified putative enhancer clusters that possibly control the target genes within a ±100 kb window, based on the Pearson correlation between gene expression and enhancer accessibility in single-cell profiles. Third, we evaluated enhancer interaction by calculating chromatin co-accessibility through Cicero (24). Enhancer pairs with significantly high co-accessibility were considered predicted enhancer interactions (PEIs). Finally, for each gene, we built an enhancer network representing how multiple enhancers interact with each other to regulate gene expression. The enhancer networks were visualized using R package igraph (v.1.2.6). Among each enhancer network, enhancers are represented as nodes, and the PEIs between enhancers are represented as edges.

### Module detection within enhancer networks

We applied the Louvain algorithm (27) for module detection using the ‘cluster_louvain’ function in the R package igraph (v1.2.6), which has been successfully utilized in Cicero for identifying *cis-*co-accessibility networks, clusters of co-accessible *cis-*elements (24).

### Defining network hub enhancers and module hub enhancers

In defining network hub enhancers, we computed the degree for each enhancer and normalized the values by accounting for the total number of nodes in the network. Enhancers with frequent PEIs in the top 5000 (approximately the 10th quantile) were identified as network hub enhancers. Similarly, for module hub enhancers, our analysis focused on PEIs within modules. We defined 5000 module hub enhancers as those displaying a notable frequency of PEIs within their respective modules.

### State/stage-specific enhancer networks

To facilitate network comparison and dynamics analysis, we constructed state/stage-specific enhancer networks in each dataset. First, we leveraged the enhancer accessibility and gene expression matrices as input for the eNet algorithm (25), which identified shared enhancer clusters (Node) for each gene across all states or stages. Following this, state/stage-specific co-accessibility between enhancers (Edge) was computed using state/stage-specific cells as input for Cicero, thereby enabling the precise construction of state/stage-specific enhancer networks.

### Identification of differential/dynamic modules during disease or development

To systematically compare enhancer networks between disease and healthy states and identify distinct modules, we drew inspiration from DiffCoEx (28), an algorithm designed to identify differentially co-expressed modules using transcriptomic data. The process involves four steps:

- *Step 1*

Build the adjacency matrix ***C*****^[^*****^n^*****^]^** for each condition *n* as the co-accessibility for all pairs of enhancers (*i, j*):


\begin{equation*}{\boldsymbol{{{C}}}^{\left[ {\boldsymbol{n}} \right]}}:{{\ }}{{{{c}}}_{{{ij}}}}^{\left[ {{n}} \right]} = {\mathrm{co}}\text{-}{\mathrm{accessbility}}\left( {{{{{E}}}_{{i}}},{{{{E}}}_{{j}}}} \right).\end{equation*}

In this step, co-accessibility was computed using Cicero (24).

- *Step 2*

Compute the adjacency difference matrix.

For two conditions:


\begin{equation*}{{\bf D}}:{{{{d}}}_{{{ij}}}} = \left| {{{{\left( {{{c}}_{{{ij}}}^{\left[ 2 \right]}} \right)}}^2} - {{{\left( {{{c}}_{{{ij}}}^{\left[ 1 \right]}} \right)}}^2}} \right|.\end{equation*}

For more than two conditions:


\begin{equation*}{{\bf D}}:\ {{{{d}}}_{{{ij}}}} = \sqrt {\frac{{\sum \left| {{{{\left( {{{{{c}}}_{{{ij}}}}^{\left[ {{n}} \right]}} \right)}}^2} - {{{\left( {{{{{c}}}_{{{ij}}}}^{\left[ 0 \right]}} \right)}}^2}} \right|}}{{{n}}}},\quad {\mathrm{where}}{{\ }}{{{{c}}}_{{{ij}}}}^{\left[ 0 \right]} = \frac{1}{{{n}}}\mathop \sum \limits_{{n}} {{{{c}}}_{{{ij}}}}.\end{equation*}

In this matrix, high values of *d_ij_* indicate significant alterations in the co-accessibility status between enhancer*_i_* and enhancer*_j_* across distinct conditions.

- *Step 3*

Derive the topological overlap-based dissimilarity matrix **T** from the adjacency change matrix **D**:


\begin{equation*}{{\bf T}}:{{{{t}}}_{{{ij}}}} = 1 - \frac{{\mathop \sum \nolimits_{{k}} ({{{{d}}}_{{{ik}}}}{{{{d}}}_{{{kj}}}}) + {{{{d}}}_{{{ij}}}}}}{{{\mathrm{min}}\left( {\mathop \sum \nolimits_{{k}} {{{{d}}}_{{{ik}}}},\mathop \sum \nolimits_{{k}} {{{{d}}}_{{{kj}}}}} \right) + 1 - {{{{d}}}_{{{ij}}}}}}.\end{equation*}

The utilization of the topological overlap measure to construct a dissimilarity matrix enables the identification of enhancers that share common neighbors in the graph formed by the differential co-accessibility network as defined by the adjacency matrix created in Step 2. In essence, a low value of *t_ij_* (high similarity) implies that both enhancer*_i_* and enhancer*_j_* exhibit significant co-accessibility changes with the same large group of enhancers.

- *Step 4*

The dissimilarity matrix **T** was used as input for clustering, and modules were identified. Standard hierarchical clustering with average linkage was employed for the clustering process, followed by module extraction from the resulting dendrogram using a fixed cut height of 0.99 by default.

### Accuracy assessment of enhancer networks

To validate the enhancer networks constructed using eNet 2.0, we performed a two-level systematic evaluation. First, at the enhancer–gene interaction level, we assessed the accuracy of eNet 2.0 by calculating the percentage of enhancer–gene pairs that overlapped with those from the widely recognized activity-by-contact (ABC) model (29), using uncorrelated enhancer–gene pairs within the same 200 kb distance as control. We further categorized genes into hematopoiesis-related and other gene groups to specifically examine the accuracy of enhancer networks associated with typical genes. Second, at the enhancer–enhancer interaction level, we employed high-resolution Hi-C data from the GM12878 and K562 cell lines, obtained from ENCODE project, as the gold standards. Enhancer pairs were classified into three categories based on Cicero scores: high (>0.2), middle (0–0.2) and low (≤0). We then calculated the percentage of enhancer pairs validated by the Hi-C data within each category.

### TF motif/binding similarity analysis

We examined the transcription factor (TF) binding on enhancers using two approaches. The first one involved DNA sequence motif analysis using Signac (v1.1.1) (30), while the second approach utilized ChIP-seq data for 361 hematopoiesis-related TFs obtained from CistromeDB (31) (Figure 2F and Supplementary Figure S1J). These two methods allowed us to generate an enhancer-TF matrix, where values of 1 and 0 denoted TF binding and non-binding to respective enhancers, respectively. Subsequently, we employed this enhancer-TF matrix to assess similarity in TF binding by calculating the cosine similarity between pairs of enhancers using the simil() function from the R package proxy (v0.4.25).

### Enrichment analysis of GWAS SNPs and FANTOM5 enhancers

The genome-wide association studies (GWAS) catalog SNPs were downloaded through the UCSC Table Browser (http://genome.ucsc.edu/). We curated a list of tissues-related GWAS SNPs using a semi-automatic text mining method (Supplementary Table S2). The enhancer RNA (eRNA) data were retrieved from FANTOM5 project through https://fantom.gsc.riken.jp/5/data/. The overlap between loci and GWAS SNPs or FANTOM5 enhancers was performed using the findOverlaps() function from the R package IRanges (v2.20.2). For enhancers in each group, the enrichment score was calculated as the fold enrichment compared to the genome background. The computing method was listed as following: (*m*/*n*)/(*M*/*N*), where *m* and *M* represent the number of SNPs or eRNA within the group and genome-wide, respectively, and *n* and *N* represent the number of loci within the group and genome-wide, respectively. The genome-wide background is generated from a list of loci obtained by randomly shuffling the list of all open chromatin regions.

### Enrichment analysis of RIC-seq interactions

We downloaded RIC-seq data in GM12878 cell line, which identified enhancer–promoter connection using pairwise interacting eRNAs and promoter-derived noncoding RNAs (32). For enhancers in each group, the enrichment score was calculated as the fold enrichment compared to the genome background. The computing method was listed as following: (*m*/*n*)/(*M*/*N*), where *m* and *M* represent the number of enhancers identified by RIC-seq that intersected with enhancers in that particular group and genome-wide, respectively, and *n* and *N* represent the number of loci within the group and genome-wide, respectively. The genome-wide background is generated from a list of loci obtained by randomly shuffling the list of all open chromatin regions.

### Synergistic effect analysis of enhancer pairs

#### Identification of enhancer-associated eQTL

To take advantage of expression quantitative trait locus (eQTL) for simulating enhancer perturbations, we first filtered gene-associated eQTL based on the significant variant–gene associations downloaded from the GTEx portal (https://gtexportal.org/). Next, for each enhancer within the enhancer network that regulates the corresponding gene, we kept the eQTL overlapped with the given enhancer as enhancer-associated eQTL.

#### The interactive model to estimate synergistic effect of paired enhancer eQTL on gene expression

To examine the synergistic effects of enhancer variants on gene expression in blood, stomach and sigmoid colon, we obtained genotype data and corresponding RNA-seq data from dbGAP (dbGaP Study Accession: phs000424.v8.p2) and the GTEx portal (https://gtexportal.org/). Utilizing each pair of enhancer-associated eQTL, we applied a linear regression model to evaluate the synergistic effect of enhancer pairs on gene expression levels through the following formula:


\begin{equation*}{Y}_{\rm {Exp}} \sim {{\beta}}_{0} + {{\beta}}_{1}{{X}}_{1}*{{X}}_{2} + {\beta}_{2}{{X}}_{1} + {\beta}_{3}{{X}}_{2} + \sum{{\beta}}_{i}*{\mathrm{Covariates}}. \end{equation*}

*X* denotes the genotype of the enhancer-associated eQTL. The selection of covariates was done following the method instruction in the previous study (33). In order to address the hidden batch effects and other technical and biological sources of variance across the transcriptome in gene expression data, we employed the probabilistic estimation of expression residual (PEER) method (34). This approach was utilized to estimate a suite of latent covariates that correspond to gene expression levels for each tissue type. The determination of the number of PEER factors was stratified across four distinct sample size categories: tissues with fewer than 150 samples, those with 150–249 samples, those with 250–349 samples and those with 350 or more samples. The optimization process, as detailed in a previous study (35), led to the selection of 15, 30, 45 and 60 PEER factors for the respective sample size bins. To mitigate the influence of population effects on the identification of quantitative trait loci (QTLs), genotype principal components (PCs) are conventionally incorporated as covariates in QTL mapping studies. Following the method instruction in the previous study (33), we included five PCs that provide an effective balance, controlling for population structure without unduly diminishing the discovery power in tissues with smaller sample sizes. Furthermore, we included covariates such as the whole genome sequencing (WGS) platform (HiSeq 2000 or HiSeq X), the WGS library construction protocol (PCR-based or PCR-free) and donor sex in our association analyses. We deem these to be the minimal essential set of covariates for most QTL mapping endeavors utilizing GTEx data. The significance of the interaction term in the fitted linear model, as indicated by the *P* value, was utilized to determine the synergistic impact of a pair of eQTL from an enhancer pair on gene expression levels. In the analysis of the human blood and colon dataset, we employed a significance threshold of *P* < 0.01 to determine synergistic variant pairs (SVPs). However, the utilization of a *P* < 0.01 threshold for the human stomach dataset led to a notably limited number of synergistic enhancer pairs (SEPs) (*n* < 100); hence, the threshold for the stomach dataset was set at *P* < 0.05. To investigate the synergistic effect at enhancer level, we defined SEPs as those with at least one SVP.

#### Comparison of synergistic and additive models

In order to ensure that the synergistic model provides a more accurate representation of the real data for the identified SVPs, we simultaneously applied an additive model without the interactive term:


\begin{equation*} {Y}_{\mathrm{Exp}} \sim {\mathrm{\beta}}_{0} + {\mathrm{\beta}}_{1}{{X}}_{1} + {\mathrm{\beta}}_{2}{{X}}_{2} + \sum {\mathrm{\beta}}_{i}*{\mathrm{Covariates}}. \end{equation*}

Then, for both the synergistic and additive models, we computed respective Akaike information criterion (AIC) scores. A lower AIC score signifies a more optimal model that effectively captures the data while considering the balance between model complexity and goodness of fit.

### Validation of enhancer interactions in MYC enhancer network

We utilized eNet 2.0 to construct the enhancer network for MYC, employing the experimentally validated seven MYC enhancers (E1–E7) from a published study as nodes (17) and Cicero-predicted chromatin co-accessibility between these enhancers as edges. Subsequently, we applied the Louvain algorithm to detect modules within the MYC enhancer network. Based on our previous findings, enhancers from different modules were predicted to interact synergistically to regulate gene expression, while enhancers within the same module contribute additively (Figure 2G–I).

To validate the synergistic/additive enhancer interactions in MYC enhancer network, we downloaded the multiplexed CRISPRi perturbation data for MYC enhancers from the original study (17). We followed the methods described in the original study to calculate depletion scores for each sgRNA pair and interaction scores for each enhancer pair. In brief, we downloaded the count matrices of sgRNA pairs at day 0 (D0) and day 30 (D30), filtered pairs with at least 30 reads in D0 and added a pseudo-count of 10. Depletion scores of all sgRNA pairs were calculated as the log_2_ enrichment scores in D30 versus D0, normalized by the mean and standard deviation of enrichment scores for control–control sgRNAs.

Next, we followed a four-step strategy, as described in the original study (17), to calculate the interaction score for each enhancer pair. First, we derived the single depletion score for a given sgRNA K by averaging the depletion scores of K-control sgRNA pairs. Second, we calculated the double depletion score for each sgRNA pair by averaging the depletion scores from both perturbations. Third, the interaction score of each query-other sgRNA pair was then calculated as the negative deviation between the observed double depletion score and the expected value derived from the linear fitting, normalized by the mean and standard deviation of the interaction scores of the query-control sgRNA pairs. Finally, we calculated the interaction score for each enhancer pair by averaging the interaction scores of the selected sgRNA pairs, which ranked in the top 50% of sgRNAs targeting the same enhancer based on their single depletion scores.

### Calculation of network score

We assigned a network score, calculated as the network connectivity, for each enhancer network in each condition during disease or development.

### Identification of network patterns during disease

To distinguish Gained/Shared/Lost networks, we compared the network scores between disease and healthy conditions. Enhancer networks ranked within the top 10% based on score difference were categorized as ‘Gained’, those within the bottom 10% as ‘Lost’ and the remaining as ‘Shared’.

### Calculation of network change score during disease

To quantify the change of enhancer network during disease, we focused solely on the altered components, and computed the network change score using the following formula:


\begin{equation*} \sum {{{c}}_{{ij}}}^{[2]} - \sum {{{c}}_{{ij}}}^{[1]}.\end{equation*}

The co-accessibility between enhancer_*i*_ and enhancer_*j*_ was denoted as ${{{{c}}}_{{{ij}}}}$, where both enhancer_*i*_ and enhancer_*j*_ were enhancers that exhibited differential co-accessibility during disease. The superscript [2] indicated disease condition, while the superscript [1] indicates healthy condition.

### Performance evaluation in predicting disease genes

To evaluate the performance of enhancer networks in predicting disease genes, we employed various scoring methods to rank all genes. These scores included network change score, network connectivity under both healthy and disease conditions, as well as their differences, SE ranks based on H3K27ac downloaded from dbSUPER database (36), gene expression variance and overall chromatin accessibility. Subsequently, we determined the fold enrichment of disease genes among the top-ranked genes using a sliding window of 100, with the entire genome serving as background.

### Identification of network dynamics patterns

To identify distinct network dynamics patterns during the differentiation or development processes, we used the network scores matrix for each enhancer network at different developmental stages as our input data. These developmental stages were categorized as Early, Middle and Late. We then converted the input matrix into a three-column matrix to represent the Early, Middle and Late stages by averaging the network scores from corresponding stages. Furthermore, enhancer networks with the highest network scores at the Early, Middle and Late stages were assigned to the Early, Middle and Late groups, respectively.

### Calculation of network dynamic score during development

To quantify the change in network score along development, we computed the network dynamic score using the following formula:


\begin{equation*} \sqrt{\frac{\sum\nolimits_{{i=1}}^{{n}}\left(\sum {{{c}}_{{ij}}}^{[{n}]} - \sum {{{c}}_{{ij}}}^{[{0}]}\right)^{2}}{{n-1}}},\quad{\mathrm{where}} \sum {{{c}}_{{ij}}}^{[{0}]} =\frac{ \sum\nolimits_{{i=1}}^{{n}}\sum {{{c}}_{{ij}}}^{[{i}]}}{{n}}. \end{equation*}

The co-accessibility between enhancer_*i*_ and enhancer_*j*_ was denoted as ${{{{c}}}_{{{ij}}}}$, where both enhancer*_i_* and enhancer_*j*_ were enhancers that exhibited dynamic co-accessibility during development, and *n* represents the number of stages.

### Retrieval of cell identity and disease genes

The cell identity genes were obtained from CellMarker database (https://www.biolegend.com/cell_markers). The disease genes were from MalaCards (https://www.malacards.org), OMIM (https://omim.org) and DisGeNET (https://www.disgenet.org/). The list of collected cell identity and disease genes can be found in Supplementary Table S3.

### Enrichment analysis of cell identity genes

In brief, given a gene group, the enrichment score was calculated as the fold enrichment relative to the genome background, determined as (*m*/*n*)/(*M*/*N*), where *m* and *M* represent the number of cell identity genes within the group and genome-wide, respectively, while *n* and *N* represent the number of genes within the group and genome-wide, respectively.

### Database construction

The current version of eNetDB was developed using MySQL 8.0 (http://www.mysql.com) and maintained on a Linux-based Nginx server. We used Python 3.8 (https://www.python.org/) and Tornado 6.1 (https://www.tornadoweb.org/) for server-side scripting, including all the interactive functions. The interactive frontend interface was designed and built using vue 2 (https://v2.vuejs.org/) and element UI (https://element.eleme.io/), which is an approachable, performant and versatile framework for building web user interfaces.

## Results

### eNet 2.0: a comprehensive tool for enhancer networks analysis

**Figure 1.**
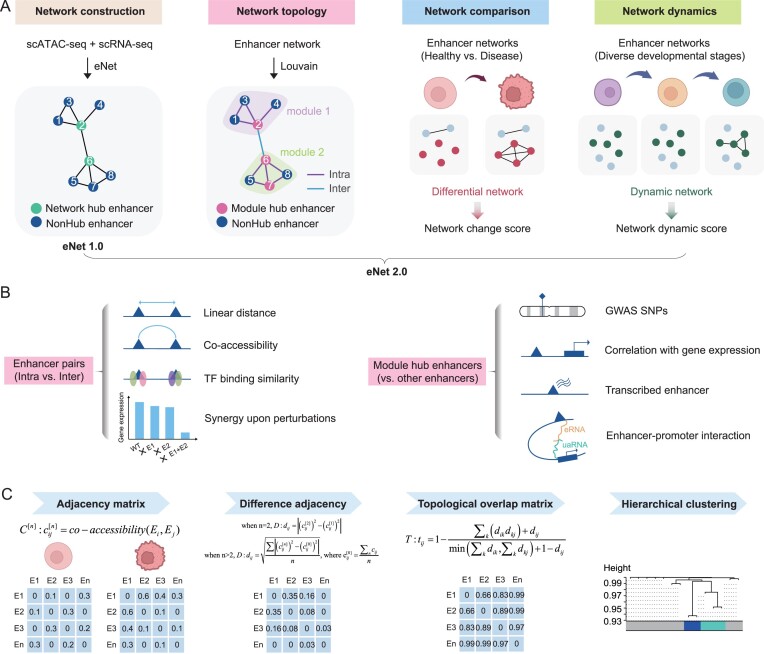
Overview of eNet 2.0. (**A**) eNet 2.0 comprises four key components for comprehensive enhancer network analysis: (i) enhancer network construction by utilizing scATAC-seq and scRNA-seq data as input, similar as eNet 1.0 (26); (ii) module identification within enhancer networks using Louvain algorithm; (iii) network comparison analysis by comparing enhancer networks from distinct conditions, such as healthy versus disease states; and (iv) network dynamics analysis by examining enhancer networks in time-series data, such as various developmental stages. (**B**) Diagram showing the characterization of (i) module-related enhancer interactions (left) and (ii) module hub enhancers (right). (i) Enhancer pairs are classified as intra (within the same module) or inter (across different modules), and are subsequently compared in terms of linear distance, chromatin co-accessibility, similarity in TF binding and synergy effects upon perturbations. (ii) Module hub enhancers are compared with other enhancers by evaluating their enrichment of GWAS SNPs, correlation with gene expression, enrichment of eRNA and enhancer–promoter interactions. (**C**) Diagram showing the processes to identify differential or dynamic modules during disease and development. The steps included (i) computing the adjacency matrix using Cicero to represent co-accessibility between enhancers for each condition, (ii) calculating the difference adjacency, denoted as **D** to quantify the overall change in co-accessibility between enhancers, (iii) deriving a topological overlap-based dissimilarity matrix, referred to as **T**, from the adjacency change matrix **D** and (iv) performing hierarchical clustering on the dissimilarity matrix **T** to identify enhancers exhibiting varying co-accessibility.

eNet 2.0 is a comprehensive tool for enhancer network analysis using scATAC-seq data. Similar to the original eNet algorithm (25), eNet 2.0 constructs enhancer networks for each gene using scATAC-seq and scRNA-seq profiles as input. An enhancer network depicts how multiple enhancers coordinate gene expression, where nodes represent enhancers and edges represent the PEIs (Figure 1A). Meanwhile, eNet 2.0 provides three additional parts for network analysis, including network topology, network comparison and network dynamics.

First, eNet 2.0 offers a wide range of functionalities for network topological analysis, including enhancer module detection, module-associated enhancer interactions and hub enhancers analysis (Figure 1A and B). Specifically, we employed the Louvain algorithm (27) to identify compact block clusters or communities within the networks, termed as modules. To determine the biological significance of the detected modules, we categorized enhancer pairs into two groups based on their source modules: Intra (pairs of enhancers from the same module) and Inter (pairs of enhancers from different modules within the same network) (Figure 1B, left). We then compared enhancer pairs within each group from several aspects, including linear distance, chromatin co-accessibility, TF binding similarity, and the propensity for synergistic or additive effects upon perturbations (Figure 1B, left). Furthermore, to investigate the hierarchy within modules, we introduced module hub enhancers, in addition to network hub enhancers in eNet 1.0 (25). To figure out whether and why module hub enhancers are functionally important, we assessed their enrichment of single-nucleotide polymorphisms (SNPs) from GWASs, correlation with gene expression, and involvement in eRNA transcription and enhancer–promoter interactions (Figure 1B, right).

Next, the third part of eNet 2.0 is differential network analysis, which aims to compare enhancer networks obtained from two different biological conditions such as disease versus normal (Figure 1A). To achieve this, we adapted DiffCoEx (28), a framework for analyzing differential co-expression using transcriptomic data, with additional modifications. In brief, starting from the adjacency matrices of enhancer networks under each condition, we quantified their differences and subsequently employed topological overlap and hierarchical clustering to identify clusters of enhancers exhibiting similar alteration patterns. The altered components represent a group of enhancers that share common neighbors in the graph formed by the differential network (Figure 1C; ‘Materials and methods’ section). We defined a network change score to quantify the magnitude of network alterations in disease and healthy states, calculated as the weighted edge degree among the altered components under disease conditions subtracted from that under healthy conditions (Figure 1A; ‘Materials and methods’ section).

Last, network dynamics analysis enables the comparison of enhancer networks in time-series data, such as various stages during tissue development (Figure 1A). The computational procedures are similar to those employed in differential network analysis, albeit with slight modifications in Step 2 (Figure 1C; ‘Materials and methods’ section). Similarly, we defined a network dynamic score to quantify the extent of changes in enhancer network across multiple developmental stages, calculated as the standard deviation of the weighted edge degree among the altered components across all stages (Figure 1A; ‘Materials and methods’ section).

Collectively, eNet 2.0 enables comprehensive analysis of enhancer networks, facilitating a deeper understanding of the intricate interplay between enhancers in gene regulation and its implications across diverse biological processes (BPs).

### Enhancer networks are modularly organized

**Figure 2.**
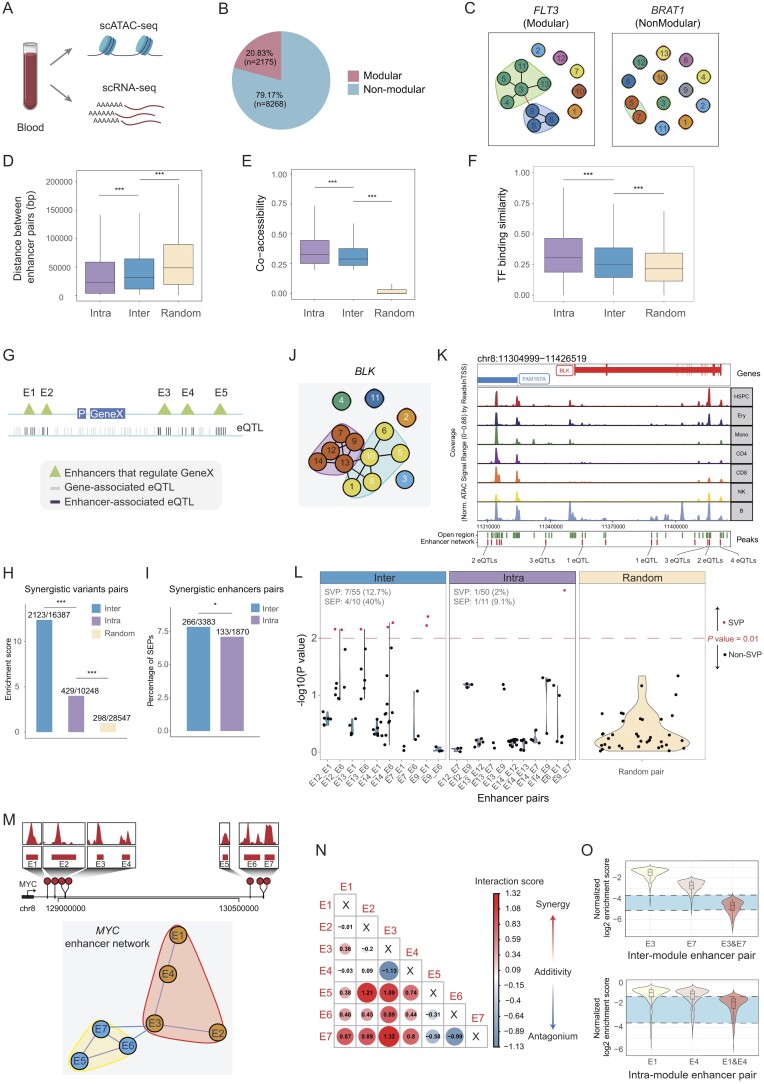
Modular organization of enhancer network provides transcriptional robustness in hematopoiesis. (**A**) The human blood dataset (37). (**B**) Pie chart showing the number and percentage of enhancer networks with modular structure (modular) or without modular structure (non-modular). (**C**) Representative modular or non-modular enhancer network. Characterization of the features of inter- and intra-module enhancer pairs, including linear distance between enhancer pairs (**D**); chromatin co-accessibility between enhancer pairs calculated using Cicero (24) (**E**), using randomly selected enhancer pairs as control. *P*-values were calculated using two-sided unpaired Student's *t*-test. *P< 0.05; **P< 0.01; ***P< 0.001; n.s., not significant. (**F**) Boxplot comparing the similarity of TF binding, quantified by cosine similarity, between inter- and intra-module enhancer pairs, using randomly selected enhancer pairs as control. *P*-values were calculated using two-sided unpaired Student's *t*-test. *P< 0.05; **P< 0.01; ***P< 0.001; n.s., not significant. (**G**) Diagram showing the method to define enhancer-associated eQTL. Gene-associated eQTL, indicated by vertical lines, are those linked to Gene X. Enhancer-associated eQTL, within the regions of a specific enhancer regulating Gene X, are also delineated. (**H**) Bar plot showing the enrichment of SVPs in different groups including Inter, Intra and Random. *P*-values were calculated using two-sided binomial test. *P< 0.05; **P< 0.01; ***P< 0.001; n.s., not significant. (**I**) Bar plot showing the percentage of SEPs in different groups including Inter and Intra. *P*-values were calculated using two-sided binomial test. *P< 0.05; **P< 0.01; ***P< 0.001; n.s., not significant. (J–L) BLK enhancer network. It comprises 14 constituent enhancers, organized into two modules (**J**). Genome browser track of BLK putative enhancer cluster, consisting of 14 enhancers, accessible in different cell types. The number of enhancer-associated eQTLs is indicated (**K**). Violin plot showing the distribution of −log_10_(*P*-value) of the synergistic model applied to variant pairs located in distinct enhancer pairs (inter and intra) from BLK enhancer networks, using random permutations as control (**L**). The number and percentage of SVPs (interactive term *P* value <0.01) and SEPs in both Inter and Intra groups were labeled in the upper left corner (**L**). (**M**) Genome browser track of MYC putative enhancer cluster, consisting of seven enhancers, accessible in K562 cells (top). The modular enhancer network of MYC (bottom). (**N** and **O**) Experimental validation of enhancer interactions adapted from Lin *et al.* (17). Heatmap depicting cell proliferation effects from multiplexed CRISPRi perturbations of various combinations of MYC enhancers E1–E7 (**N**). Representative examples of enhancer pair interactions: inter-modular pair (E3 and E7) demonstrating synergistic effects on cell proliferation (top), and intra-modular pairs (E1 and E4) showing additive effects (bottom). The regions bounded by dashed lines indicate the expected additive effects based on individual enhancer perturbations (**O**).

Compared with eNet, the first feature of eNet 2.0 is network module analysis, which included enhancer module detection, inter-/intra-module enhancer interactions and hub enhancers analysis. To test these functions, we applied eNet 2.0 on a hematopoiesis dataset reported in a broadly cited literature (37) (Figure 2A). In total, eNet 2.0 identified 10 443 enhancer networks, involving 109 482 PEIs among 64 792 enhancers. The size of these networks ranged from 1 to 101, with a median size of 4 (Supplementary Figure S1A). The accuracy of these enhancer networks was confirmed at both enhancer–gene and enhancer–enhancer interaction levels, using the ABC model and Hi-C data as gold standards (see ‘Materials and methods’ section). Notably, we observed particularly high enhancer–gene mapping accuracy (47.1%) for hematopoiesis-related genes (Supplementary Figure S1B), and a consistent pattern between PEIs and Hi-C data (Supplementary Figure S1C and D). Network topology analysis identified 2175 (20.83%) modular networks, which contained at least two modules within enhancer networks (Figure 2B). Most modular networks contained two modules, with each module typically comprising 2–27 enhancers (Supplementary Figure S1E). Notably, genes regulated by modular enhancer networks showed higher expression levels and cell-type specificity compared to those regulated by non-modular networks (Supplementary Figure S1F and G). In addition, these modular network-regulated genes were enriched in hematopoiesis-related pathways, while non-modular network-regulated genes were associated with basic cellular pathways such as cell cycle (Supplementary Figure S1H). For instance, the enhancer network controlling FLT3, a crucial cytokine receptor in hematopoiesis, displayed a modular organization with two distinct modules (Figure 2C, left). In contrast, BRAT1, which regulates cell cycle checkpoint signaling, showed a non-modular network structure (Figure 2C, right).

Next, to understand the biological significance of the detected modules, we compared inter- and intra-module enhancer interactions. First, we quantified the linear distance between enhancer pairs within each group and observed a significantly higher linear proximity within the Intra group (Figure 2D). Additionally, we noted that the enhancer pairs in Intra group exhibited higher co-accessibility scores as well as mutual information than Inter group (Figure 2E and Supplementary Figure S1I). To investigate the involvement of TFs in gene regulation, we first quantified the similarity of TF motifs on enhancer pairs using cosine similarity (see ‘Materials and methods’ section). The Intra group displayed higher similarity in TF motifs on enhancer pairs compared to the Inter group, using the Random group as background (Supplementary Figure S1J). Additionally, we curated ChIP-seq data for 361 human blood-related TFs from CistromeDB (31). We evaluated the similarity of TF binding on enhancer pairs of the two groups, and observed a consistent trend where intramodular enhancer pairs displaying higher similarity of TF binding than intermodular pairs (Figure 2F). Overall, these findings suggested that enhancer networks are organized into modules, where enhancers within each module are close in linear distance and highly interactive, sharing similar TF binding profiles.

### Inter-module enhancer interactions exhibit synergistic effects on gene expression

A recent study reveals a nested epistasis enhancer network for robust gene expression regulation using multiplexed CRISPRi perturbation of ultralong-distance enhancers at the MYC locus (17). Inspired by this finding, we next evaluated whether genetic variants on inter-module enhancer pairs, which span spatial separation, can alter gene regulation in a synergistic manner. To achieve this, we downloaded eQTL data for human blood samples from the GTEx portal (https://www.gtexportal.org/), leveraged eQTL variants within enhancers (enhancer-associated eQTL) to simulate the perturbation of corresponding enhancers, and fitted a linear regression model with an interaction term to analyze the combined effects of perturbing an enhancer pair on gene expression (Figure 2G; ‘Materials and methods’ section). Our analysis uncovered that inter-module enhancer variants interacted more frequently to alter target gene expression than intra-module enhancer variants (Supplementary Figure S1K). Further, we defined SVPs as those that synergistically alter gene expression by assessing the significance of the interaction term (see ‘Materials and methods’ section). We calculated the enrichment score of SVPs in three groups (Inter, Intra and Random). Interestingly, the Inter group exhibited the highest enrichment of SVPs (Figure 2H). Additionally, for each SVP, we calculated an AIC score to determine if the synergistic model fitted better compared to the additive model, with a lower AIC score indicates better fitness (see ‘Materials and methods’ section). As expected, our analysis revealed that the AIC score of the synergistic model was lower than that of the additive model (Supplementary Figure S1L).

Next, we extended this analysis to the enhancer level by defining SEPs as those containing at least one SVP, and calculating the percentage of SEPs in each group (see ‘Materials and methods’ section). We found a significantly higher proportion of SEPs in Inter group than Intra group (Figure 2I). An illustration from our analysis is the regulation of *BLK*, a gene encoding a crucial player in B-cell receptor signaling and development, by an enhancer network consisting of 14 elements arranged into two modules (Figure 2J). Notably, seven of these enhancers (E1, E6, E7, E9, E12, E13 and E14) harbored at least 1 eQTL, totaling 16 eQTLs (Figure 2K). These eQTLs formed 105 pairwise combinations, with 55 pairs belonging to the Inter group and 50 to the Intra group. Interestingly, at the variant level, we identified 7 SVPs out of 55 enhancer pairs (12.7%) in the Inter group, contrasting with only 1 SVP among 50 pairs (2%) in the Intra group (Figure 2L). At the enhancer level, we identified 4 SEPs out of 10 enhancer pairs (40%) in the Inter group, whereas only 1 SEP was detected among 11 pairs (9.1%) in the Intra group (Figure 2L). These results indicated that the inter-modular enhancer pairs, which mainly enriched SEPs, did exert a synergistic influence on gene expression.

To further validate our results, we applied eNet 2.0 to analyze the enhancer network consisting of seven enhancers (namely E1–E7) regulating MYC, where both additive and synergistic effect between enhancers have been observed through multiplexed CRISPRi screen experiments (17). Our analysis of the MYC enhancer network revealed two distinct modules: one comprising enhancers E1–E4 and another containing enhancers E5–E7 (Figure 2M). Based on our earlier findings (Figure 2G–I), enhancers from different modules were predicted to interact synergistically to regulate gene expression, while enhancers within the same module would demonstrate additive effects. Strikingly, utilizing the multiplexed CRISPRi screen data generated by Lin *et al.* (17), we confirmed this observation that enhancers from different modules contributed synergistically to cell proliferation, while enhancers within the same module (e.g. E1–E4 or E5–E7) showed additive effects (Figure 2N; ‘Materials and methods’ section). For example, individual perturbations of enhancers E1, E3, E4 or E7 had mild impact on cell proliferation. However, simultaneous disruption of inter-module enhancer pairs (e.g. E3 and E7) dramatically reduced cell growth, whereas concurrent perturbation of intra-module pairs (e.g. E1 and E4) showed only additive effects (Figure 2O). These observations aligned precisely with the findings from the original study (17). These results not only validated the accuracy of eNet 2.0 but also underscored the critical importance of enhancer networks in gene regulation and cell viability.

Together, our findings suggested that enhancer modules, which were spatially separated, contributed to the robustness of enhancer networks against perturbations.

### Module hub enhancers are functionally important

**Figure 3.**
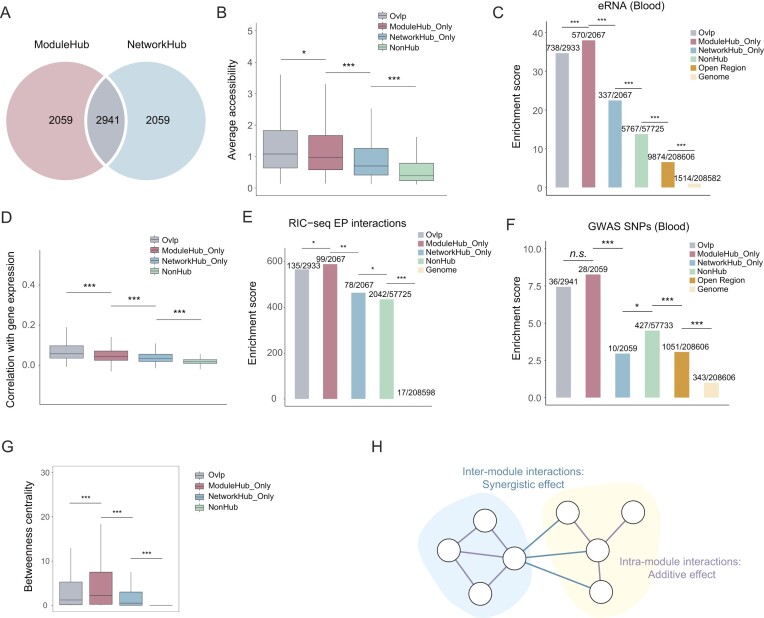
Module hub enhancers are functionally important. (**A**) Venn plot showing two types of hub enhancers (module hub enhancers and network hub enhancers) and their overlap (Ovlp enhancers). (**B**) Boxplot showing the average chromatin accessibility of enhancers across various groups: Ovlp, ModuleHub_Only, NetworkHub_Only and NonHub. *P*-values were calculated using two-sided unpaired Student's *t*-test. *P< 0.05; **P< 0.01; ***P< 0.001; n.s., not significant. (**C**) Enrichment of eRNA from FANTOM5 project (38) in different groups of enhancers including Ovlp, ModuleHub_Only, NetworkHub_Only and NonHub, using the whole genome as the background. *P*-values were calculated using the two-sided binomial test. *P< 0.05; **P< 0.01; ***P< 0.001; n.s., not significant. (**D**) Boxplot showing the correlation between enhancers and target genes across various groups: Ovlp, ModuleHub_Only, NetworkHub_Only and NonHub. *P*-values were calculated using two-sided unpaired Student's *t*-test. *P< 0.05; **P< 0.01; ***P< 0.001; n.s., not significant. (**E**) Enrichment of enhancer–promoter pairs from RIC-seq data (32) across various groups: Ovlp, ModuleHub_Only, NetworkHub_Only and NonHub, using the whole genome as the background. *P*-values were calculated using the two-sided binomial test. *P< 0.05; **P< 0.01; ***P< 0.001; n.s., not significant. (**F**) Enrichment of blood-related GWAS SNPs in different groups of enhancers including Ovlp, ModuleHub_Only, NetworkHub_Only and NonHub, using the whole genome as the background. *P*-values were calculated using the two-sided binomial test. *P< 0.05; **P< 0.01; ***P< 0.001; n.s., not significant. (**G**) Boxplot comparing the betweenness centrality of enhancers across various groups: Ovlp, ModuleHub_Only, NetworkHub_Only and NonHub. *P*-values were calculated using two-sided unpaired Student's *t*-test. *P < 0.05; **P < 0.01; ***P < 0.001; n.s., not significant. (**H**) Model of modular enhancer network.

To delve deeper into the hierarchy of each module within enhancer networks, we introduced the concept of module hub enhancers, distinct from the network hub enhancers in our previous study (25,26) (Figure 1A; ‘Materials and methods’ section). In the human blood dataset, we identified 5000 enhancers for both module hub and network hub, with 2941 enhancers found to overlap between the two sets, referred to as ‘Ovlp’ (Figure 3A and Supplementary Figure S2A). Comprehensive characterization revealed that module hub enhancers possess distinct features compared to network hub and non-hub enhancers (Supplementary Table S4). These features included closer linear distance to promoter (Supplementary Figure S2B), increased chromatin accessibility (evidenced by scATAC-seq, bulk ATAC-seq and DNase-seq data, H3K4me1 ChIP-seq signals) (Figure 3B and Supplementary Figure S2C), stronger enhancer activity (demonstrated by eRNA levels and H3K27ac ChIP-seq signals) (Figure 3C and Supplementary Figure S2C), and more robust correlation with gene expression (supported by single-cell and RIC-seq data) (Figure 3D and E). Notably, module hub enhancers showed significant higher enrichment for GWAS SNPs, particularly those linked to hematopoiesis, highlighting their functional significance in gene regulation (Figure 3F and Supplementary Figure S2D). To elucidate the roles and potential mechanisms of different hub enhancers in gene regulation, we analyzed their network properties and interactions. Module hub enhancers exhibited the highest betweenness centrality, indicating their function as critical network bridges (Figure 3G). Furthermore, module hub-associated inter-module enhancer pairs showed the strongest tendency toward synergistic interactions (Supplementary Figure S2E and F), highlighting their pivotal role in facilitating enhancer synergy.

Collectively, these findings reveal that enhancer networks possess not only modular organization with biological significance, but also a hierarchical structure within individual modules, where module hub enhancers play critical roles (Figure 3H). This hierarchical organization provides new insights into the complexity of enhancer-mediated gene regulation.

### Alterations of enhancer network correlate with abnormal gene expression in disease

**Figure 4:**
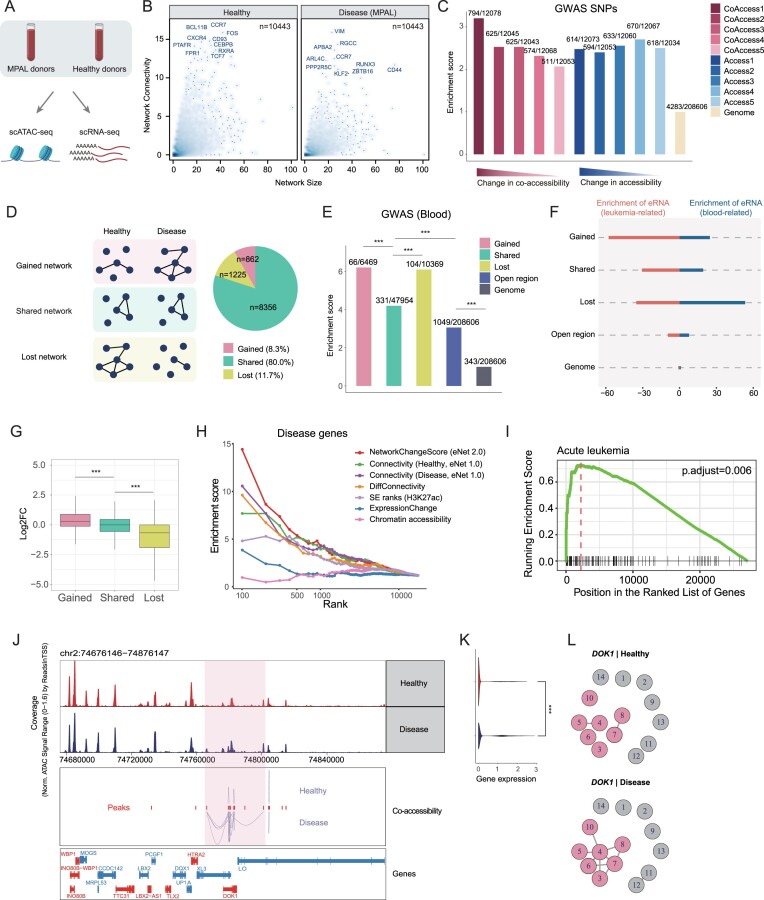
eNet 2.0 identifies differential enhancer networks in MPAL. (**A**) The MPAL dataset, including single-cell multi-omics profiles from bone marrow and peripheral blood mononuclear cells from MPAL patients and healthy donors (37). (**B**) Scatter density plots showcasing the enhancer networks in blood samples from healthy (left) and MPAL (right), with the *x*-axis displaying the network size and the *y*-axis indicating network connectivity. Top 10 genes ranked by network connectivity are labeled. (**C**) Bar plot showing the enrichment of GWAS SNPs in diverse groups ordered by the change of chromatin co-accessibility as well as accessibility between healthy and MPAL groups. *P*-values were calculated using binomial test. *P< 0.05; **P< 0.01; ***P< 0.001; n.s., not significant. (**D**) Diagram showing the definitions of Gained/Shared/Lost enhancer networks (left), and the pie chart (right) displaying the percentages of these three types of enhancer networks. Bar plot showing the enrichment of blood-related GWAS SNPs (**E**) and leukemia-/blood-related eRNA (**F**) across various groups including Gained, Shared and Lost, using the whole genome as background. *P*-values were calculated using two-sided binomial test. *P< 0.05; **P< 0.01; ***P< 0.001; n.s., not significant. (**G**) Boxplot showing the log_2_FC distribution of gene expression in leukemia patients compared to healthy donors within different groups: Gained, Shared and Lost. *P*-values were calculated using two-sided unpaired Student's *t*-test. *P< 0.05; **P< 0.01; ***P< 0.001; n.s., not significant. (**H**) Enrichment of hematopoiesis-related disease genes (*y*-axis) for the top-ranked genes ordered by several metrics measured by (i) network change score, (ii) network connectivity under healthy conditions, (iii) network connectivity under disease conditions, (iv) the difference in network connectivity comparing disease to healthy condition, (v) SE ranks based on H3K27ac signals calculated by ROSE (4), (vi) ExpressionChange comparing the gene expression between disease and healthy conditions and (viii) overall chromatin accessibility. (**I**) The significantly enriched disease ontology associated with acute leukemia determined through GSEA analysis based on network change score. (**J**) Genome browser track of DOK1 putative enhancer cluster, consisting of 14 enhancers, accessible under healthy (top) and disease (bottom) conditions, with 7 differential enhancers in co-accessibility highlighted. (**K**) Violin plot showing DOK1 relative expression under healthy (top) and disease (bottom) conditions. *P*-values were calculated using two-sided unpaired Student's *t*-test. *P< 0.05; **P< 0.01; ***P< 0.001; n.s., not significant. (**L**) Enhancer network regulating DOK1 under healthy (top) and disease (bottom) conditions, with seven enhancers in Gained network highlighted.

The second feature in eNet 2.0 is the network comparison analysis part, which aims to compare enhancer networks obtained from two different biological conditions. To this end, we applied it to the single-cell transcriptomic and chromatin landscapes of bone marrow and peripheral blood mononuclear cells from both mixed-phenotype acute leukemia (MPAL) patients and healthy donors (37) (Figure 4A). In total, we built 10 443 enhancer networks in MPAL and healthy donors, respectively (Figure 4B; ‘Materials and methods’ section). To test whether the changes of enhancer networks might be disease-relevant, we first assessed GWAS SNP enrichment among enhancers ranked based on changes of chromatin co-accessibility. Interestingly, we found the changes of chromatin co-accessibility of enhancer interactions showed a clear correlation with GWAS SNP enrichment (Figure 4C, left). Notably, this association was not evident when classifying enhancers solely based on changes in chromatin accessibility (Figure 4C, right). To further validate these findings, we identified differentially expressed genes (DEGs) and differentially accessible regions (DARs) by comparing MPAL patients with healthy donors. We categorized DEGs into two groups: Disease (blood-related disease genes) and Other genes. Analysis of DAR distribution showed that over 70% of disease-associated genes were associated with multiple DARs, a significantly higher proportion compared to other genes (Supplementary Figure S5A). These findings indicated that significant changes in disease-related gene expression are typically driven by the coordinated regulation of multiple enhancers, rather than by individual enhancers acting alone.

To systematically identify the differential enhancer networks during disease progression, we compared enhancer networks in MPAL to healthy donors. It resulted in a total of 862 Gained, 1225 Lost and 8356 Shared enhancer networks (Figure 4D; ‘Materials and methods’ section). The enhancers within Gained and Lost enhancer networks displayed higher enrichment of hematopoiesis-related GWAS SNPs compared to those in Shared enhancer networks (Figure 4E). Moreover, leukemia-associated eRNA were mainly enriched in Gained enhancer networks, while normal hematopoiesis-related eRNA were mainly enriched in Lost enhancer networks (Figure 4F). We next examined the changes in gene expression to investigate whether the alterations of enhancer networks correlate with abnormal gene expression. To this end, we conducted a DEG analysis using matched scRNA-seq data, and compared the log_2_ fold change (log_2_FC) values of genes controlled by three modes including Gained, Shared and Lost. Remarkably, the Gained group exhibited the highest log_2_FC value, while the Lost group showed the lowest log_2_FC value (Figure 4G). These observations strongly suggested that changes in enhancer networks were closely associated with aberrant gene expression, indicating their potential role in driving abnormal gene expression in disease.

### Enhancer network change score in eNet 2.0 predicts disease genes

Next, we sought to investigate whether alterations in enhancer networks during disease progression, as measured by network change score, can prioritize genes associated with blood-related diseases. To test this, we ranked enhancer networks according to several metrics: the network change score calculated via eNet 2.0; the network connectivity—a feature previously demonstrated in eNet 1.0 (25) to correlate with disease-linked genes—evaluated separately for diseased and healthy conditions, alongside the discrepancy between the two states; additionally, we incorporated SE ranks based on H3K27ac signal, as well as ExpressionChange obtained from DEG analyses, employing overall chromatin accessibility levels as control (see ‘Materials and methods’ section). Subsequently, we curated blood-related disease genes from the MalaCards database and assessed the enrichment of these genes within the list of top-ranked genes related to enhancer networks based on distinct scores, using the entire genome as background (Figure 4H; ‘Materials and methods’ section). We found that DEGs, which compared disease with healthy conditions, can partially identify disease-related genes. Notably, SE ranks outperformed DEGs, underscoring the additional insights provided by epigenetic analysis. In addition, the assessment of network connectivity in healthy/disease states and their disparities can also aid in the identification of disease genes and outperformed SE, suggesting that the interactions between enhancers provided additional information than enhancer clusters. Most importantly, the network change score (eNet 2.0) demonstrated the best performance with 14.4-fold enrichment in top 100 genes, superior to original eNet 1.0 (25) and other methods (Figure 4H). Moreover, the gene set enrichment analysis (GSEA) based on network change score revealed the significant enrichment of the disease ontology associated with acute leukemia (Figure 4I). These findings underscored the pivotal roles of altered enhancer networks in driving abnormal gene expression, particularly in disease-related genes. For instance, in our analysis, the *DOK1* gene, implicated in leukemia, showed a regulatory network of 14 enhancers (Figure 4J and L). Of note, while no observable difference in chromatin accessibility was detected between the two states, a significant discrepancy was noted in chromatin co-accessibility (Figure 4J). Our network comparison analysis revealed that, compared with the healthy condition, *DOK1* gained a distinct differential enhancer network involving seven enhancers in MPAL (Figure 4L). Concurrently, DEG analysis confirmed its significant up-regulation in MPAL (Figure 4K).

To understand the superior performance of NetworkChangeScore, we compared the top 500 genes identified by these seven different metrics. Our analysis revealed that metrics based on network connectivity showed substantial gene overlap with each other, while differential expression-based metrics ExpressionChange shared few genes with other metrics (Supplementary Figure S5B). Particularly, when comparing NetworkChangeScore and ExpressionChange, we found these two methods identified distinct sets of genes, with only 11 overlapping genes (Supplementary Figure S5C).

We found genes identified by NetworkChangeScore showed higher change of network connectivity, but minor changes on gene expression, comparing with DEGs (Supplementary Figure S5D and E). We speculated NetworkChangeScore tend to identify upstream regulators with key regulatory roles, whereas mRNA profiling frequently detects downstream targets with dramatic expression changes. Indeed, genes with high NetworkChangeScore are typically regulated by a greater number of enhancers and show higher enrichment in TFs curated from JASPAR database (Supplementary Figure S5F and G). Despite their significant enhancer network changes, these genes often do not exhibit dramatic expression changes themselves (Supplementary Figure S5D and E).

These findings collectively demonstrate that our network comparison analysis identifies key regulatory genes that might be ignored by conventional differential expression analysis, providing crucial insights into disease progression through the lens of enhancer networks.

### Dynamics of enhancer networks ensure precise temporal expression during development

**Figure 5.**
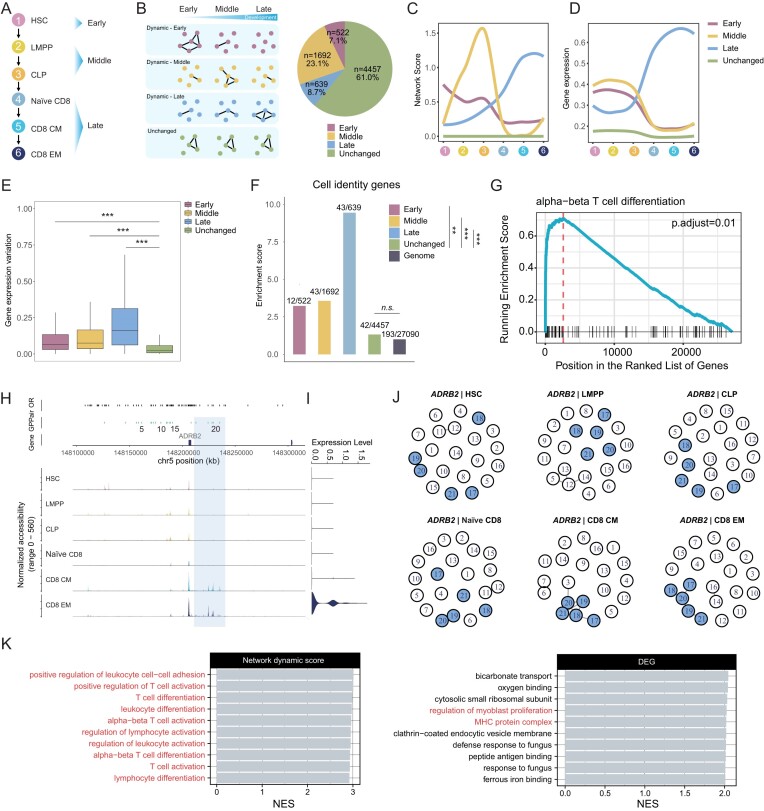
eNet 2.0 identifies dynamic enhancer networks along CD8^+^ T-cell differentiation. (**A**) Diagram illustrating the differentiation path from HSC to LMPP, CLP, naïve CD8, CD8 CM and finally to CD8 EM cells. The stages are categorized as Early (HSC), Middle (LMPP and CLP), and Late (naïve CD8, CD8 CM and CD8 EM). (**B**) Diagram (left) showing the definitions of stage-specific networks (including Early, Middle and Late), Unchanged network across cellular differentiation, and the pie chart (right) displaying the percentages of these four groups. The curve displaying the fitted network score (**C**) and gene expression (**D**) of enhancer networks within diverse groups (Early, Middle, Late and Unchanged) across six stages as depicted in panel (**A**). (**E**) Boxplot showing the distribution of gene expression variation across stages within distinct groups (Early, Middle, Late and Unchanged). *P*-values were calculated using two-sided unpaired Student's *t*-test. *P< 0.05; **P< 0.01; ***P< 0.001; n.s., not significant. (**F**) Enrichment of CD8^+^ T-cell-related cell identity genes in different groups (Early, Middle, Late and Unchanged), using the whole genome as background. *P*-values were calculated using the two-sided binomial test. *P< 0.05; **P< 0.01; ***P< 0.001; n.s., not significant. (**G**) The top-ranked enriched BP pathway identified via GSEA based on network change score. (**H**) Enhancer network regulating ADRB2 along CD8^+^ T-cell differentiation, with five enhancers in differential modules highlighted. (**I**) Violin plot showing ADRB2 relative expression in each of the six stages shown in (**A**) along CD8^+^ T-cell differentiation. (**J**) Enhancer network regulating ADRB2 along CD8^+^ T-cell differentiation, with five dynamic enhancers highlighted. (**K**) Top 10 enriched BP pathways as determined by gene set enrichment analysis (GSEA), based on network change score (left) and log_2_FC value of DEGs (right), with immune-related pathways highlighted.

To evaluate the network dynamics analysis part of eNet 2.0, we applied it to a differentiation trajectory spanning six stages, from hematopoietic stem cells (HSC) to lymphoid-primed multipotent progenitor (LMPP), common lymphoid progenitor (CLP), naïve CD8 T cell (naïve CD8), CD8 central memory T cell (CD8 CM) and ultimately to CD8 effector memory T (CD8 EM) cells (37) (Figure 5A). A total of 7310 enhancer networks were constructed for each stage along this trajectory. Subsequently, we contrasted the enhancer networks across the differentiation stages, identifying dynamic enhancer networks by examining the presence of dynamic constituents within each network. This led to the identification of 2853 dynamic and 4457 unchanged enhancer networks (Figure 5B; ‘Materials and methods’ section).

To further differentiate distinct dynamic patterns, we categorized all dynamic enhancer networks into three groups based on their network scores: (i) ‘Early’ denoting networks exhibiting the highest network score in the early stage (HSC), (ii) ‘Middle’ representing networks with the highest network score in the intermediate stages (LMPP and CLP) and (iii) ‘Late’ indicating networks with the highest network score in the late stages (naïve CD8, CD8 CM and CD8 EM) (Figure 5A and B; ‘Materials and methods’ section). This classification yielded 1688 Early, 1692 Middle and 639 Late dynamic networks (Figure 5B). We fitted a curve depicting the dynamics of network scores during cell differentiation for enhancer networks within each group, which showed consistent dynamic patterns in line with our definition (Figure 5C and Supplementary Figure S7A).

Next, we aimed to link distinct dynamic patterns of enhancer networks to gene expression. To achieve this, we calculated the average gene expression values of each enhancer network at each stage, followed by fitting smooth curves to the average expression values of the six stages for each group. Notably, our analysis revealed a high degree of synchronicity between the dynamics of enhancer networks and alterations in gene expression (Figure 5C and D, and Supplementary Figure S7A and B). Moreover, it was observed that the three dynamic groups (Early, Middle and Late) exhibited higher expression levels as well as greater expression variation compared to the unchanged group (Figure 5E). Additionally, the dynamic groups demonstrated higher enrichment of CD8^+^ T-cell-related cell identity genes than unchanged group (Figure 5F). For instance, we observed an enhancer network associated with *ADRB2*, the marker of effector CD8^+^ memory T (CD8 EM) cells, composed of 21 enhancers (Figure 5H and J). Notably, network dynamics analysis revealed a unique dense sub-network involving five enhancers at the Late stages (CD8 CM and CD8 EM), which preceded high *ADRB2* expression in CD8 EM cells (Figure 5I and J). This finding suggested that dynamics of enhancer networks may serve as a predictive indicator for changes in gene expression.

Next, we calculated a network dynamic score to quantify the degree of alterations for each dynamic enhancer network throughout differentiation (Figure 1A; ‘Materials and methods’ section). To further investigate the biological significance of these changes, we conducted GSEA. Our analysis revealed a significantly enriched term ‘alpha–beta T-cell differentiation’, which was closely linked to the function of CD8^+^ T cells (Figure 5G). Intriguingly, the GSEA results using the network dynamic score revealed that all of the top 10 enriched BP terms were associated with immunity (Figure 5K, left). In contrast, when we performed GSEA using the log_2_FC values derived from DEG analysis, only two immunity-related terms were found to be enriched (Figure 5K, right). These results indicated that changes in enhancer networks offer a more comprehensive insight compared to gene-level analysis, aiding in the identification of key genes or pathways involved in differentiation.

Together, these results highlighted the dynamic nature of enhancer networks regulating key genes, driving precise temporal expression during development.

### eNet 2.0 is robust and broadly applicable

To comprehensively assess the broad applicability of the three primary functions in eNet 2.0, we applied it across a diverse range of tissue types and biological contexts.

**Figure 6:**
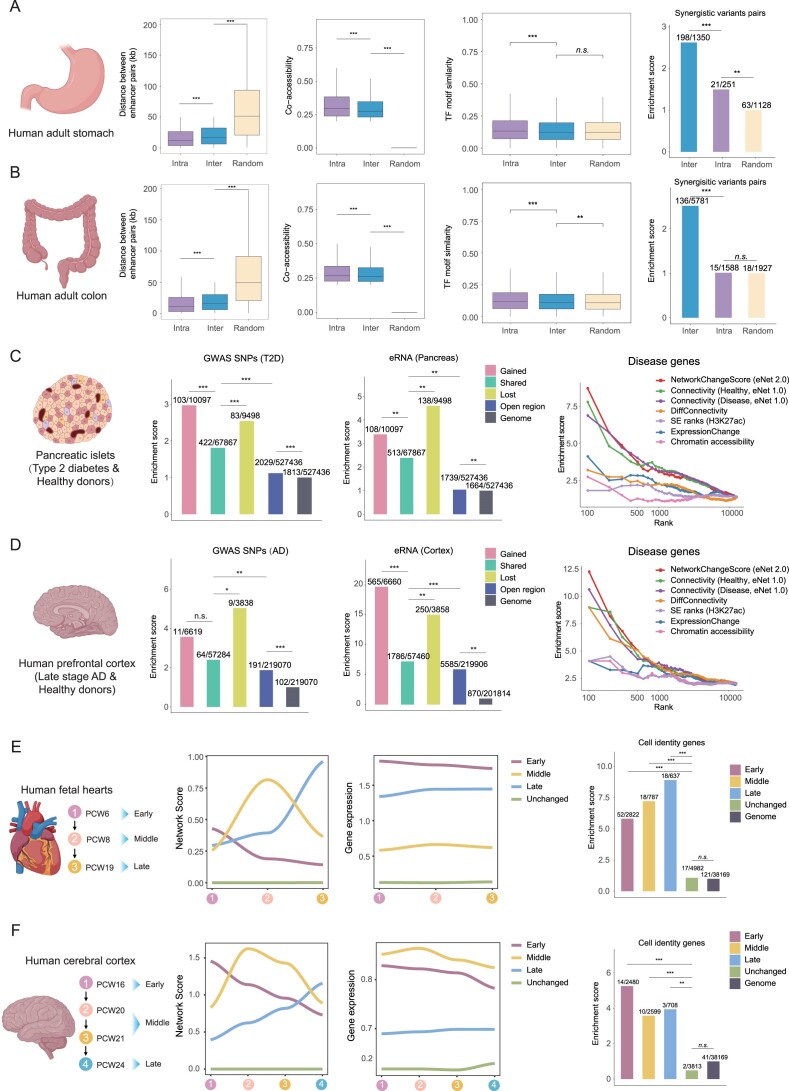
eNet 2.0 is robust across various tissues and biological contexts. Linear distance, chromatin co-accessibility and TF motif similarity between enhancer pairs, as well as enrichment of SEPs from diverse groups (Inter, Intra and Random) in human adult stomach (**A**) and colon (**B**) datasets, respectively. *P*-values were calculated using two-sided unpaired Student's *t*-test and the two-sided binomial test. *P< 0.05; **P< 0.01; ***P< 0.001; n.s., not significant. Enrichment of GWAS SNPs, disease-specific eRNA and disease-related genes in human T2D (**C**) and AD (**D**) datasets, respectively. *P*-values were calculated using the two-sided binomial test. *P< 0.05; **P< 0.01; ***P< 0.001; n.s., not significant. Dynamics of enhancer network and gene expression, and enrichment of cell identity genes in developing human fetal hearts (**E**) and human cerebral cortex (**F**) datasets, respectively. *P*-values were calculated using the two-sided binomial test. *P< 0.05; **P< 0.01; ***P< 0.001; n.s., not significant.

First, to rigorously evaluate whether modular organization of enhancer network exists across various biological contexts, we analyzed two additional publicly available scATAC-seq datasets, including human adult stomach and colon (39). Similar to the findings in hematopoiesis, we identified around 10% networks with modular organization, featuring linear proximity, higher co-accessibility and interdependence, and similar TF motifs (Figure 6A and B, and Supplementary Figures S3A and B and S4A and B). Importantly, inter-module enhancer pairs consistently exhibited synergistic effects on gene regulation (Figure 6A and B, and Supplementary Figures S3C and D and S4C and D). Furthermore, hub enhancers showed higher chromatin accessibility, strong correlations with gene expression, as well as higher enrichment of GWAS SNPs (Supplementary Figures S3E–I and S4E–I). Taken together, we provided robust evidence supporting the widespread existence of functionally significant modular organization within enhancer networks, highlighting the susceptibility of hub enhancers to diseases due to their close ties to gene expression.

Next, in order to assess the robustness of network comparison analysis, we conducted parallel analyses to identify differential enhancer networks and their biological significance in type 2 diabetes (T2D) (40) and Alzheimer’s disease (AD) (41). Consistent with the findings in MPAL dataset, we noted a distinct correlation between the alterations in enhancer interactions and the enrichment of GWAS SNPs, which was not evident for chromatin accessibility (Supplementary Figure S6A and C). Additionally, ∼10% of enhancer networks exhibited gained or lost activity throughout disease progression, wherein the enhancers displayed higher enrichment of disease-relevant GWAS SNPs and tissue-related eRNA (Figure 6C and D, and Supplementary Figure S6B and D). Notably, the network change score demonstrated exceptional performance in predicting disease genes (Figure 6C and D). Collectively, these findings underscored the correlation between altered enhancer networks and disease genes throughout disease progression.

Last, to evaluate the robustness of network dynamics analysis, we obtained scATAC-seq data for the developing human fetal heart at post-conceptional weeks (PCW) 6, 8 and 19 (42), and human cerebral cortex at PCW 16, 20, 21 and 24 (43). Similar to the findings in CD8^+^ T-cell differentiation, ∼50% of enhancer networks exhibited dynamic activity throughout heart and brain development, aligning with the dynamics of gene expression (Figure 6E and F, and Supplementary Figure S7C and E). Moreover, these dynamic networks exhibited significantly higher enrichment of cell identity genes (Figure 6E and F). GSEA results indicated that the network dynamic score prioritized more tissue-related pathways compared to DEG analysis, thereby providing strong validation of the efficacy and reliability of our method in pinpointing genes relevant to cell fate determination (Supplementary Figure S7D and F).

In summary, eNet 2.0 showed robustness in analyzing enhancer networks across diverse biological contexts, spanning tissue types, disease states and developmental stages.

### eNetDB to explore enhancer networks in various human and mouse tissues

**Figure 7:**
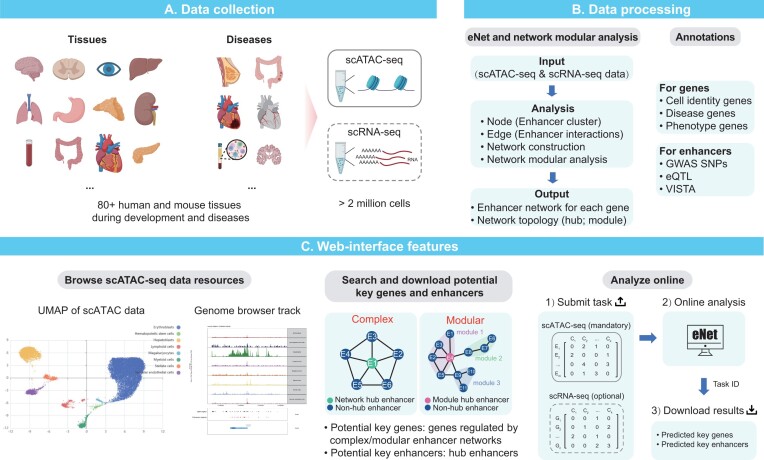
eNetDB overview. eNetDB (**A**) collected the existing over 80 scATAC-seq datasets across diverse human and mouse tissues during development and diseases, (**B**) performed eNet and network modular analysis on each dataset to construct enhancer networks and predicted key genes and enhancers, with additional genomic and epigenomic annotations, and (**C**) provided a user-friendly interface that allows users to browse, search, download and analyze the predicted key genes and enhancers.

Given the evidence supporting the pivotal role of enhancer networks in elucidating gene regulation mechanisms, there is an urgent need for a comprehensive enhancer network database. To address this, we established eNetDB (https://enetdb.huanglabxmu.com), a database containing 80 high-quality scATAC-seq datasets (Figure 7A and Supplementary Table S1). The datasets were derived from different single-cell sequencing platforms, including 10X genomics (https://www.10xgenomics.com/), SHARE-seq (44), SNARE-seq (22), sci-ATAC-seq3 (45) and sci-ATAC-seq (46) (Supplementary Figure S8A). Among these datasets, 44 were derived from human samples and 36 from mouse samples (Supplementary Figure S8B). Furthermore, we categorized these datasets into two groups: tissues (comprising 72 datasets) and diseases (comprising 8 datasets), representing samples from healthy tissues and patients, respectively (Supplementary Figure S8C). We identified key genes and enhancers in various human and mouse tissues, integrating genomic annotations that include known associations such as cell identity, diseases and phenotypes (Figure 7B). Furthermore, we incorporated GWAS SNPs, eQTL and experimentally validated VISTA enhancers to provide comprehensive enhancer annotations (Figure 7B). Our platform not only allows users to browse public scATAC-seq datasets and access predicted key genes and enhancers, but also offers an online eNet analysis module for customizing single-cell chromatin accessibility data (Figure 7C). Overall, eNetDB is a comprehensive and user-friendly platform for gene and enhancer research in human and mouse development and diseases.

## Discussion

In this study, we presented eNet 2.0, a comprehensive toolkit for enhancer network analysis using scATAC-seq data. This new version significantly advances beyond our previous work on eNet 1.0, both in methodology and in biological findings. Methodologically, eNet 2.0 maintains the core capability of constructing enhancer networks from scATAC-seq data while introducing three pivotal new modules: network topology, network comparison and network dynamics analysis (Figure 1A). Biologically, eNet 2.0 reveals two major advances. First, it effectively identifies biologically relevant functional modules within enhancer networks. Second, through differential and dynamic network analyses, it transcends the limitations of eNet 1.0 that were confined to static network analysis and unable to capture the dynamic nature of epigenetic regulation during development and disease. Collectively, these improvements provided deeper understanding of epigenetic regulation in developmental processes and disease progression.

The introduction of modular organization within enhancer networks addressed challenges faced in elucidating the functional significance of individual enhancers, which often do not exhibit significant phenotypic effects upon deletion (47). This long-standing issue is, in part, due to a limited comprehension of enhancer interactions, such as synergy, additivity or redundancy. While Lin *et al.* illustrated synergy between enhancers through multiplexed CRISPR screening, their approach is hampered by low throughput and high costs, and they primarily relied on several disease-related genes as case studies to illustrate the synergistic interactions between enhancers spanning ultra-long genomic distances (17). In contrast, our research extended this phenomenon to the genome scale, by leveraging rich and readily available single-cell multi-omics data to delve into the modular structure of enhancer networks, revealing a tendency for synergistic interactions between enhancer pairs from different modules (Figures 2G–I and 6A and B, and Supplementary Figures S3C and S4C). Further investigation of network hierarchy uncovered a hierarchical organization both globally and within modules. Our model distinguishes two types of hub enhancers: NetworkHub enhancers, characterized by high degree centrality, maintain the global network's structural integrity, while ModuleHub enhancers, with high betweenness centrality, facilitate inter-module communication and synergistic interactions (Supplementary Table S4). This modular arrangement ensures robust local control through intra-module additive effects while enabling precise gene expression through inter-module synergy (Figure 3H). Our findings shed light on the cooperative nature among enhancers and emphasize the importance of considering their interactions when investigating their functional roles.

In comparison to the three-dimensional enhancer networks, which often rely on Hi-C-based technologies (48), our approach using scATAC-seq data provides a higher resolution view of enhancer interactions, allowing us to capture the intricate and dynamic interactions within enhancer networks with greater fidelity. Benefit from this, our network comparison and dynamics analyses effectively and robustly predicted disease genes and cell identity genes, outperforming traditional SEs, DEG analysis (49) and our original method eNet 1.0 (25,26) (Figures 4H, 5K, and 6C and D, and Supplementary Figure S7D and F). The richer insights obtained from enhancer data over gene expression data, combined with the limitations of eNet in providing causal relationship, underscored the effectiveness of eNet 2.0. Overall, the changes and dynamics in enhancer–enhancer interactions significantly improved our understanding of disease progression and developmental decisions.

While eNet 2.0 shows impressive performance in detecting biologically significant modules, identifying differential and dynamic enhancer networks, and revealing epigenetic insights in the context of disease and development, there is still potential for improvement. First, relying solely on the correlation between chromatin accessibility and gene expression at the single-cell level may lead to potential errors. To improve accuracy, future updates could consider integrating high-resolution Hi-C data or employing advanced machine learning methods. Additionally, further *in vitro* and *in vivo* experimental validations are crucial to deepen our understanding of the regulatory roles of enhancer clusters under real biological contexts.

In conclusion, eNet 2.0 represents a tool for the comprehensive analysis of enhancer networks through the integration of single-cell multi-omics data. It holds the potential to advance precision medicine by facilitating the identification of genes and enhancers linked to diseases and development, as well as revealing intrinsic regulatory mechanisms that could offer valuable insights for therapeutic interventions.

## Supplementary Material

gkae1323_Supplemental_Files

## Data Availability

The single-cell datasets utilized for eNet 2.0 and eNetDB analysis were curated from published studies, as listed in Supplementary Table S1. The ABC model for assessing the accuracy of eNet 2.0 was download from https://mitra.stanford.edu/engreitz/oak/public/Nasser2021/AllPredictions.AvgHiC.ABC0.015.minus150.ForABCPaperV3.txt.gz. The MYC multiplexed CRISPRi screen data were obtained from published study GSE160768. Processed data for H3K27ac, H3K4me1 ChIP-seq, DNase-seq, ATAC-seq and Hi-C were downloaded from the ENCODE project, with all accession IDs listed in Supplementary Table S1. eNetDB is freely accessible for users through the link (https://enetdb.huanglabxmu.com) without registration or login. The code for eNet 2.0 analysis is available on GitHub (https://github.com/xmuhuanglab/eNet2.0), which is linked to Zenodo (DOI: 10.5281/zenodo.13349432).
