# Supplementary material for: Modular organization of enhancer network provides transcriptional robustness in mammalian development
Source: Nucleic Acids Res. 2025 Jan 16;53(2):gkae1323. doi: 10.1093/nar/gkae1323 (PMC11736433; doi:10.1093/nar/gkae1323)
Supplement: gkae1323_Supplemental_Files [file gkae1323_supplemental_files.zip › SupplementaryFigures1-8.pdf]

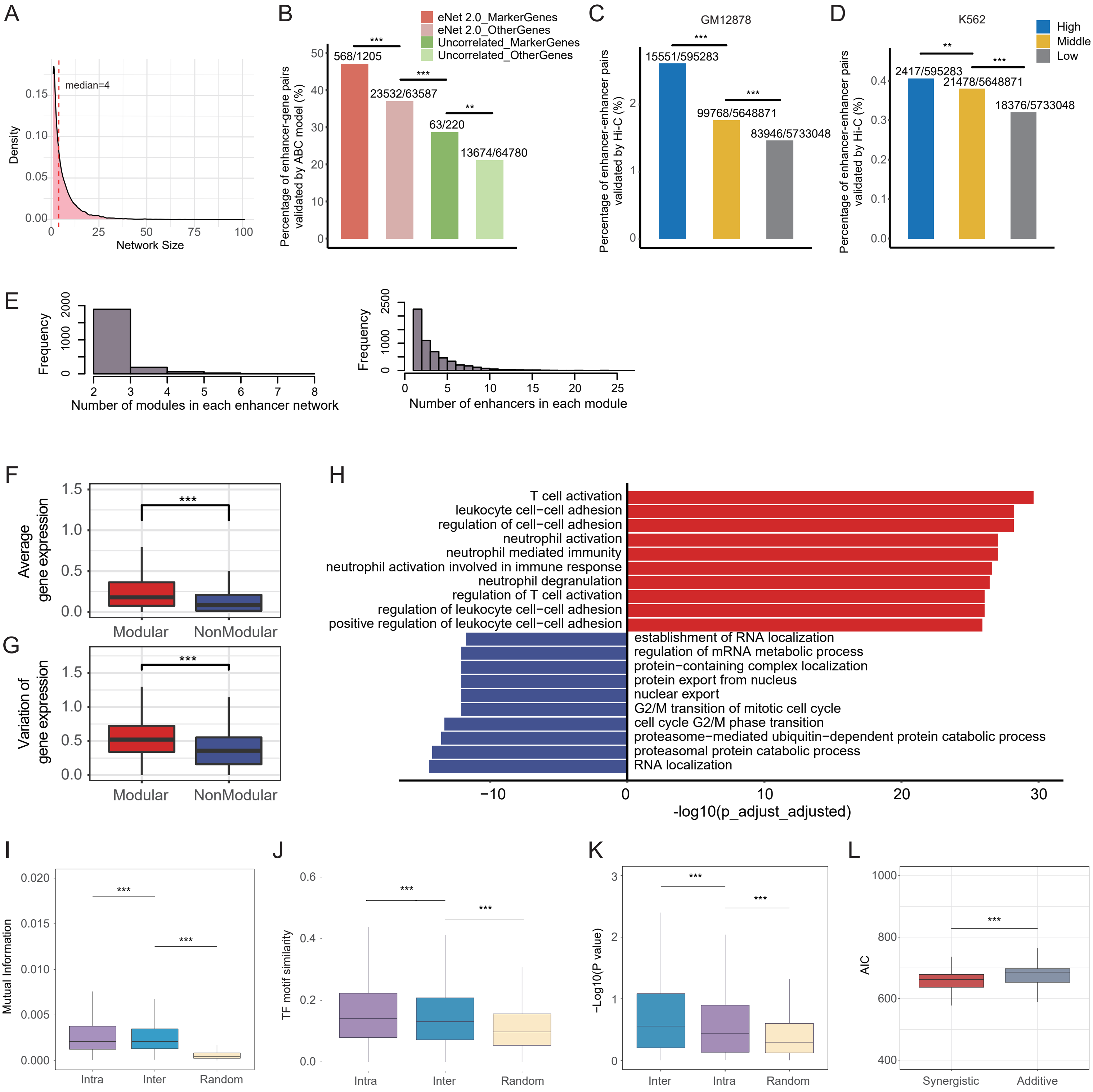

Supplementary Figure S1. Network module analysis in human blood dataset. Related to **Figure 2**. (**A**) Density plot showing the distribution of network size. (**B**) Bar plots comparing the percentages of validated enhancer-gene pairs by the ABC model. *P*-values are calculated using two-sided binomial test. *$P < 0.05$; **$P < 0.01$; ***$P < 0.001$; *n.s.*, not significant. (**C** and **D**) Bar plot showing the percentage of validated enhancer-enhancer pairs by Hi-C in GM12878 (**C**) and K562 (**D**) cells. *P*-values are calculated using two-sided binomial test. *$P < 0.05$; **$P < 0.01$; ***$P < 0.001$; *n.s.*, not significant. (**E**) Histogram showing the frequency of module number in each enhancer network (left) and enhancer number in each module (right). (**F** and **G**) Boxplot showing the average gene expression (**F**) and expression variation (**G**) between genes regulated by modular and non-modular enhancer networks. *P*-values are calculated using two-sided unpaired Student's t-test. *$P < 0.05$; **$P < 0.01$; ***$P < 0.001$; *n.s.*, not significant. (**H**) Barplot showing the Gene Ontology (GO) enrichment analysis for genes regulated by modular and non-modular enhancer networks. (**I**) Boxplot showing the mutual information between enhancer pairs across diverse groups (Inter, Intra and Random). *P*-values were calculated using two-sided unpaired Student's t-test. *$P < 0.05$; **$P < 0.01$; ***$P < 0.001$; *n.s.*, not significant. (**J**) Boxplot showing the TF motif similarity between enhancer pairs across diverse groups (Inter, Intra and Random), as quantified by cosine similarity. *P*-values were calculated using two-sided unpaired Student's t-test. *$P < 0.05$; **$P < 0.01$; ***$P < 0.001$; *n.s.*, not significant. (**K**) Boxplot showing the distribution of -log10(*P* value) evaluating the synergistic effects of enhancer pairs on gene expression across various groups (Inter, Intra, and Random). *P*-values were calculated using two-sided unpaired Student's t-test. *$P < 0.05$; **$P < 0.01$; ***$P < 0.001$; *n.s.*, not significant. (**L**) Boxplot showing the AIC score for synergistic and additive model, respectively. *P*-values were calculated using one-sided paired Student's t-test. *$P < 0.05$; **$P < 0.01$; ***$P < 0.001$; *n.s.*, not significant.

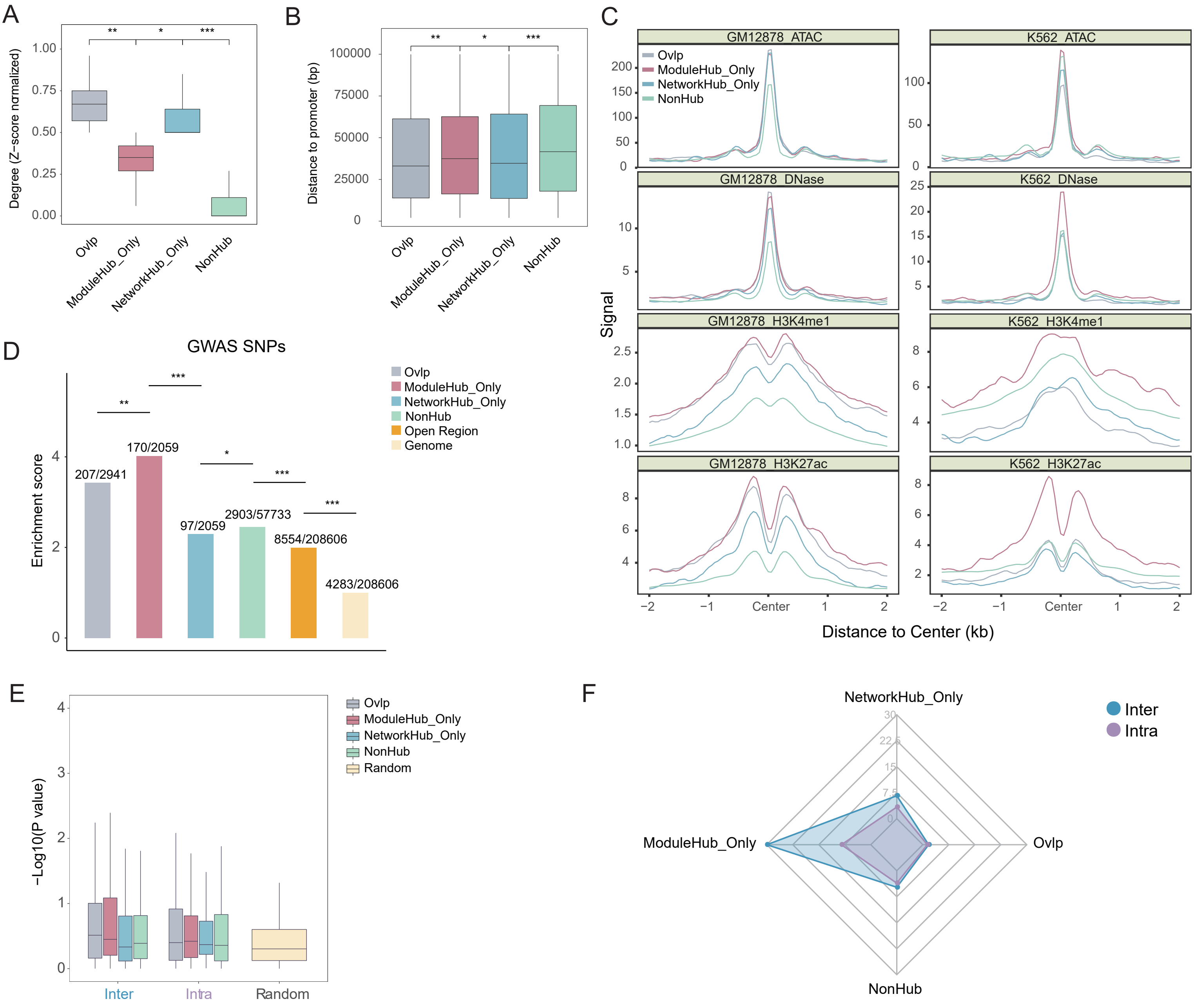

Supplementary Figure S2. Hub enhancer analysis in human blood dataset. Related to **Figure 3**. (**A**) Boxplot showing the z-score normalized degree in different groups (Ovlp, ModuleHub_Only, NetworkHub_Only and Nonhub). *P*-values were calculated using two-sided Student's t-test. **P* < 0.05; ***P* < 0.01; ****P* < 0.001; *n.s.*, not significant. (**B**) Boxplot showing the distribution of distances from different types of enhancers (Ovlp, ModuleHub_Only, NetworkHub_Only, and NonHub) to promoters. *P*-values were calculated using two-sided unpaired Student's t-test. **P* < 0.05; ***P* < 0.01; ****P* < 0.001; *n.s.*, not significant. (**C**) Distribution of ATAC, DNase, H2K4me1 and H3K27ac signals within a 4-kb window cantered around different types of enhancers (Ovlp, ModuleHub_Only, NetworkHub_Only, and NonHub) in GM12878 (top) and K562 (bottom) cells. (**D**) Enrichment of all GWAS SNPs in different groups of enhancers (Ovlp, ModuleHub_Only, NetworkHub_Only and Nonhub), using the whole genome as the background. *P*-values were calculated using the two-sided binomial test. **P* < 0.05; ***P* < 0.01; ****P* < 0.001; *n.s.*, not significant. (**E**) Boxplot showing the distribution of -log10(*P* value) evaluating the synergistic effects of enhancer pairs on gene expression in different groups, initially categorized as Inter or Intra, followed by the presence of specific enhancer types, including: 1) Ovlp (enhancer pairs containing at least one Ovlp enhancer), 2) ModuleHub_Only (enhancer pairs with at least one module hub enhancers), 3) NetworkHub_Only (enhancer pairs with at least one network hub enhancers), and 4) NonHub (enhancer pairs without any hub enhancers). (**F**) Radar plot demonstrating the enrichment of SVP within the same eight distinct groups as shown in (**E**).

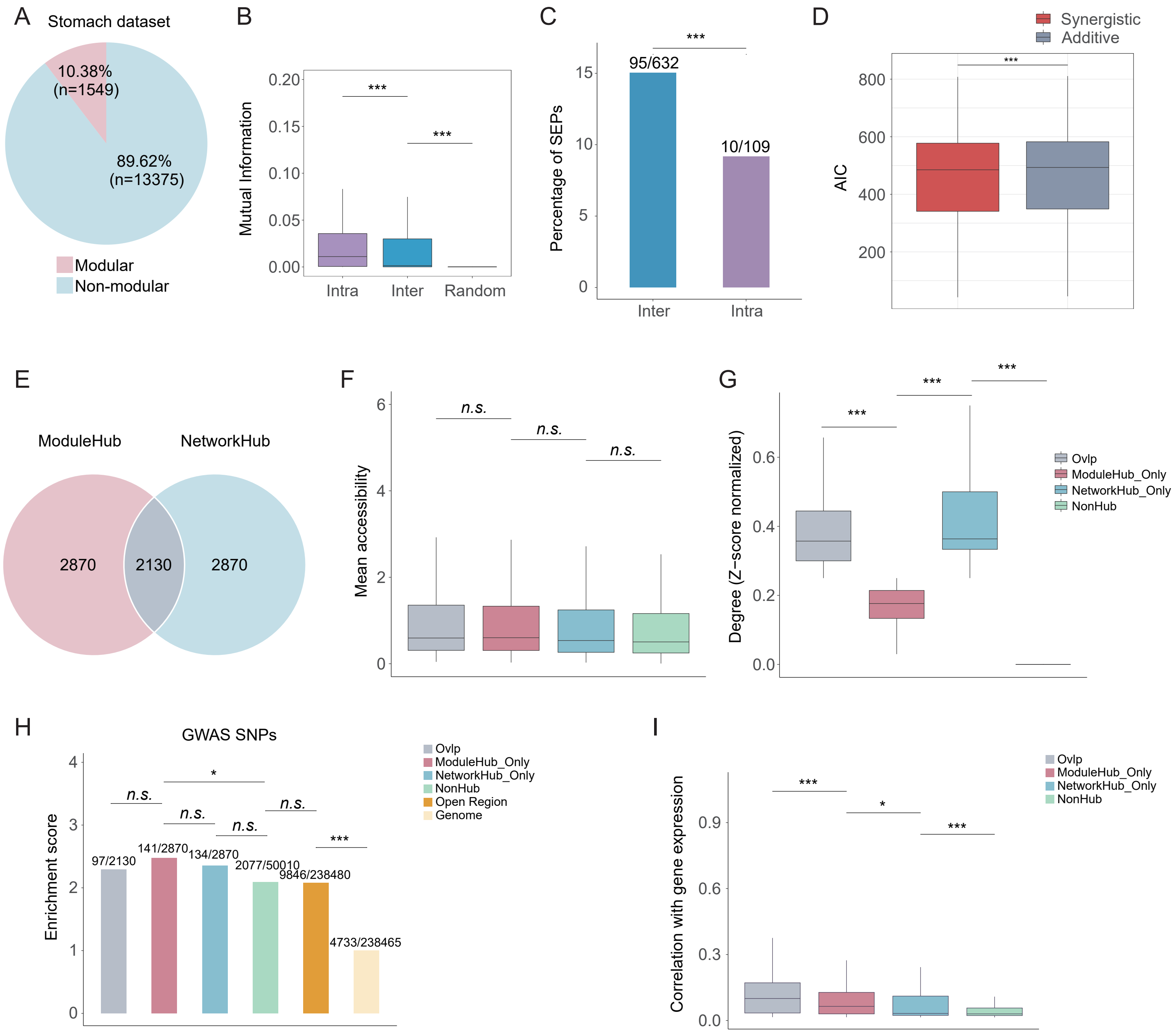

Supplementary Figure S3. Network module analysis in human stomach dataset. Related to **Figure 6**. (**A**) Pie chart showing the number and percentage of enhancer networks in the stomach dataset, categorizing them into those with modular structure (Modular) and those without (Non-modular). (**B**) Boxplot showing the mutual information between enhancer pairs across diverse groups (Inter, Intra and Random). *P*-values are calculated using two-sided unpaired Student's t-test. **P* < 0.05; ***P* < 0.01; ****P* < 0.001; *n.s.*, not significant. (**C**) Bar plot showing the percentage of synergistic enhancer pairs in different groups including Inter and Intra. *P*-values were calculated using two-sided binomial test. **P* < 0.05; ***P* < 0.01; ****P* < 0.001; *n.s.*, not significant. (**D**) Boxplot showing the AIC score for synergistic and additive model, respectively. *P*-values were calculated using one-sided paired Student's t-test. **P* < 0.05; ***P* < 0.01; ****P* < 0.001; *n.s.*, not significant. (**E**) Venn plot showing the overlap of the two types of hub enhancers (module hub enhancers and network hub enhancers) and their overlap (Ovlp enhancers). (**F**) Boxplot showing the average chromatin accessibility in different groups (Ovlp, ModuleHub_Only, NetworkHub_Only and Nonhub). *P*-values were calculated using two-sided unpaired Student's t-test. **P* < 0.05; ***P* < 0.01; ****P* < 0.001; *n.s.*, not significant. (**G**) Boxplot showing the z-score normalized degree in different groups (Ovlp, ModuleHub_Only, NetworkHub_Only and Nonhub). *P*-values were calculated using two-sided unpaired Student's t-test. **P* < 0.05; ***P* < 0.01; ****P* < 0.001; *n.s.*, not significant. (**H**) Enrichment of GWAS SNPs in different groups of enhancers including Ovlp, ModuleHub_Only, NetworkHub_Only, and NonHub, using the whole genome as the background. *P*-values were calculated using the two-sided binomial test. **P* < 0.05; ***P* < 0.01; ****P* < 0.001; *n.s.*, not significant. (**I**) Boxplot showing the correlation between enhancers and target genes across various groups: Ovlp, ModuleHub_Only, NetworkHub_Only, and NonHub. *P*-values were calculated using two-sided unpaired Student's t-test. **P* < 0.05; ***P* < 0.01; ****P* < 0.001; *n.s.*, not significant.

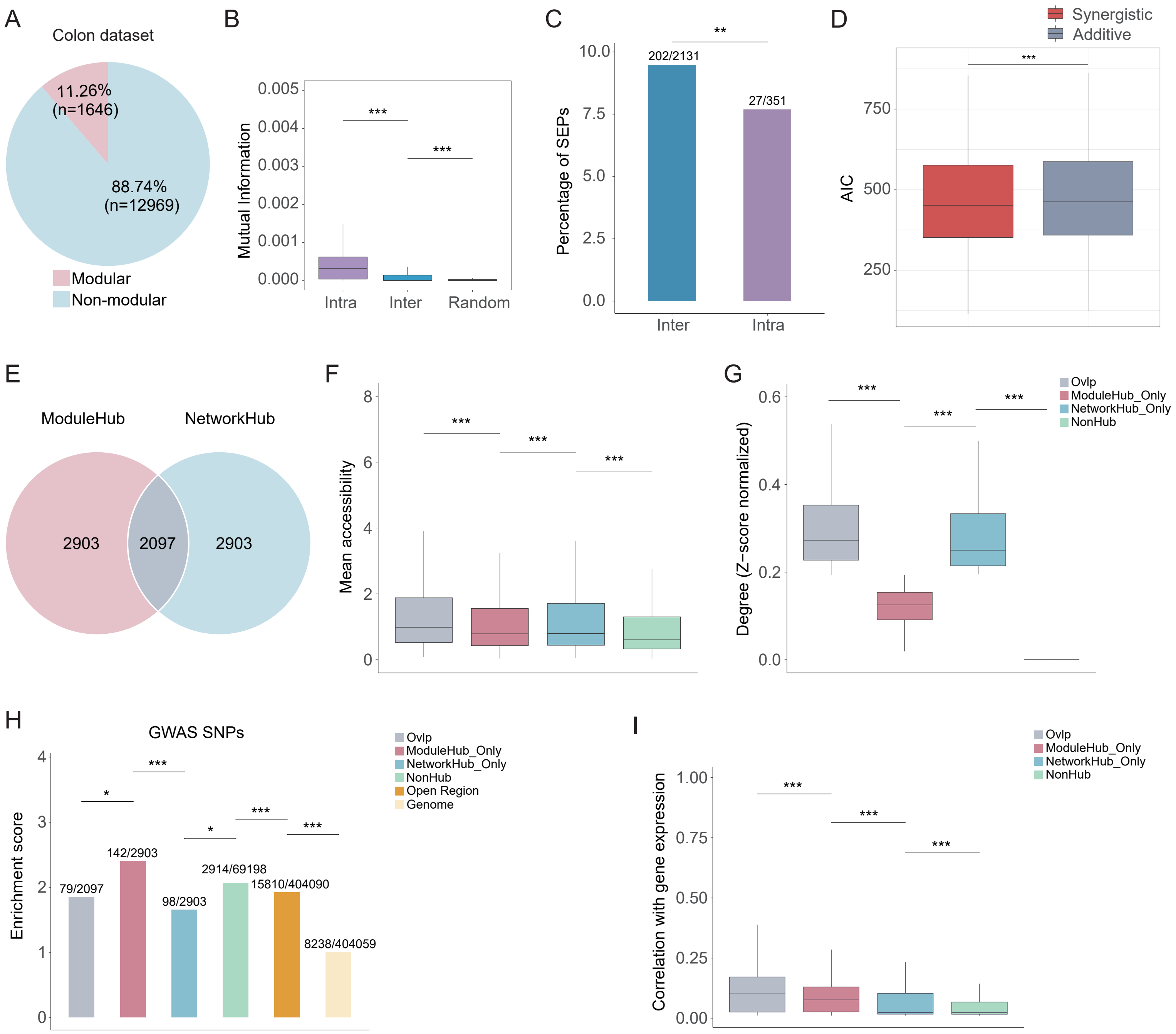

Supplementary Figure S4. Network module analysis in human colon dataset. Related to **Figure 6**. (**A**) Pie chart showing the number and percentage of enhancer networks in the colon dataset, categorizing them into those with modular structure (Modular) and those without (Non-modular). (**B**) Boxplot showing the mutual information between enhancer pairs across diverse groups (Inter, Intra and Random). *P*-values are calculated using two-sided unpaired Student's t-test. **P* < 0.05; ***P* < 0.01; ****P* < 0.001; *n.s.*, not significant. (**C**) Bar plot showing the percentage of synergistic enhancer pairs in different groups including Inter and Intra. *P*-values were calculated using two-sided binomial test. **P* < 0.05; ***P* < 0.01; ****P* < 0.001; *n.s.*, not significant. (**D**) Boxplot showing the AIC score for synergistic and additive model, respectively. *P*-values were calculated using one-sided paired Student's t-test. **P* < 0.05; ***P* < 0.01; ****P* < 0.001; *n.s.*, not significant. (**E**) Venn plot showing the overlap of the two types of hub enhancers (module hub enhancers and network hub enhancers) and their overlap (Ovlp enhancers). (**F**) Boxplot showing the average chromatin accessibility in different groups (Ovlp, ModuleHub_Only, NetworkHub_Only and Nonhub). *P*-values were calculated using two-sided unpaired Student's t-test. **P* < 0.05; ***P* < 0.01; ****P* < 0.001; *n.s.*, not significant. (**G**) Boxplot showing the z-score normalized degree in different groups (Ovlp, ModuleHub_Only, NetworkHub_Only and Nonhub). *P*-values were calculated using two-sided unpaired Student's t-test. **P* < 0.05; ***P* < 0.01; ****P* < 0.001; *n.s.*, not significant. (**H**) Enrichment of GWAS SNPs in different groups of enhancers including Ovlp, ModuleHub_Only, NetworkHub_Only, and NonHub, using the whole genome as the background. *P*-values were calculated using the two-sided binomial test. **P* < 0.05; ***P* < 0.01; ****P* < 0.001; *n.s.*, not significant. (**I**) Boxplot showing the correlation between enhancers and target genes across various groups: Ovlp, ModuleHub_Only, NetworkHub_Only, and NonHub. *P*-values were calculated using two-sided unpaired Student's t-test. **P* < 0.05; ***P* < 0.01; ****P* < 0.001; *n.s.*, not significant.

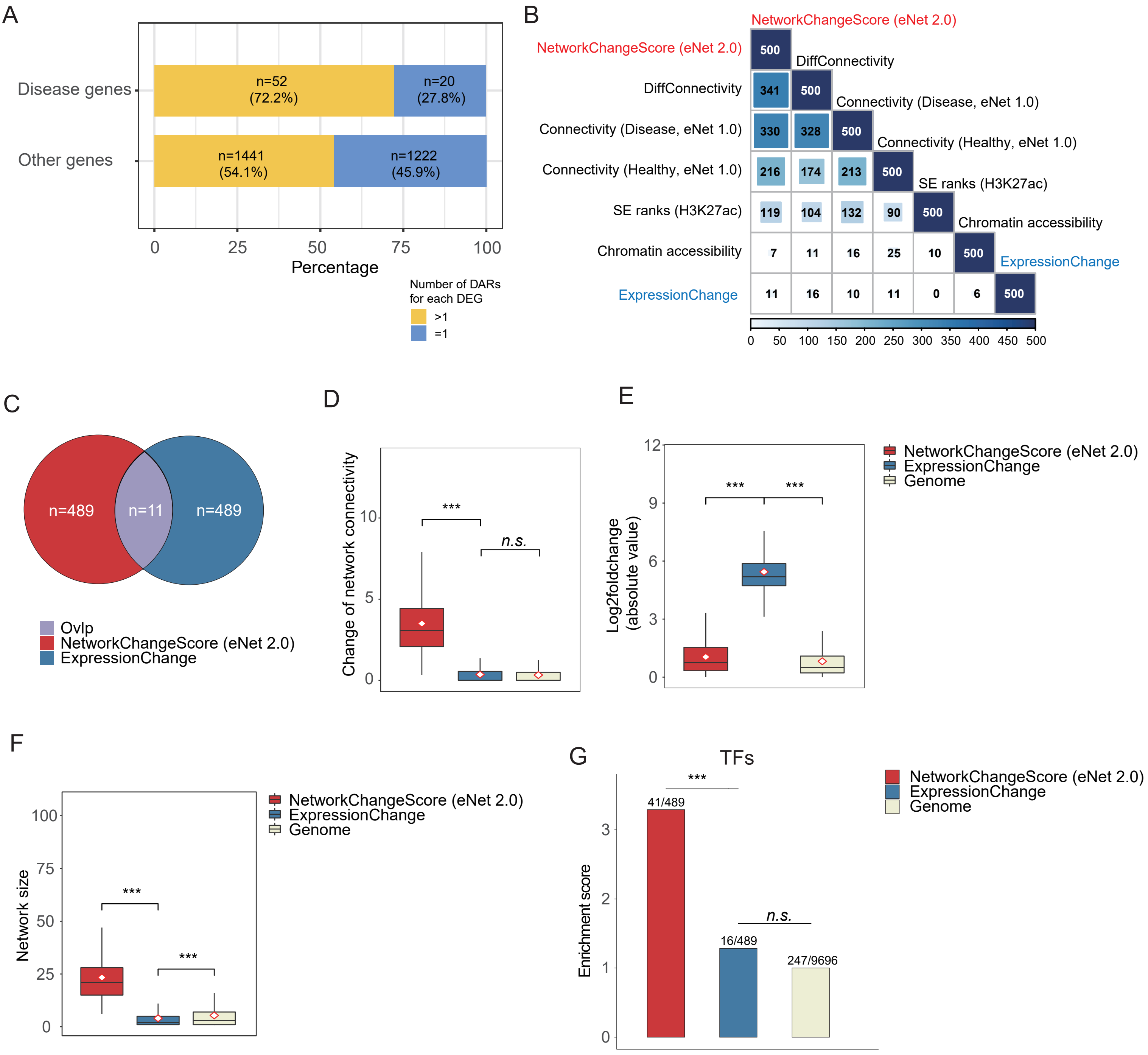

Supplementary Figure S5. Network comparison analysis in MPAL dataset. Related to **Figure 4**. (**A**) The stacked bar chart illustrates the proportion of differentially expressed genes (DEGs) that are regulated by either a single differential enhancer (blue) or multiple differential enhancers (yellow) across different categories of DEGs, including leukemia-related disease genes and other genes. (**B**) Heatmap depicting the intersection of the top 500 genes based on several connectivity metrics: NetworkChangeScore (eNet 2.0), DiffConnectivity, Connectivity (Disease, eNet 1.0), Connectivity (healthy, eNet 1.0), SE ranks (H3K27ac), chromatin accessibility, and ExpressionChange. The numbers within the heatmap indicate the count of genes shared between the metrics. (**C**) Venn diagram showing the overlap between top 500 genes identified by NetworkChangeScore (eNet 2.0) and ExpressionChange. (**D-F**) Boxplot comparing the difference of network connectivity (**D**), the absolute value of log2foldchange (**E**) and network size (**F**) across various groups of genes identified by NetworkChangeScore (eNet 2.0) and ExpressionChange, using genome as control. *P*-values were calculated using two-sided unpaired Student's t-test. *$P < 0.05$; **$P < 0.01$; ***$P < 0.001$; *n.s.*, not significant. (**G**) Barplot displaying the enrichment score of transcription factors (TFs) within each group (NetworkChangeScore, ExpressionChange, and Genome). *P*-values are calculated using two-sided binomial test. *$P < 0.05$; **$P < 0.01$; ***$P < 0.001$; *n.s.*, not significant.

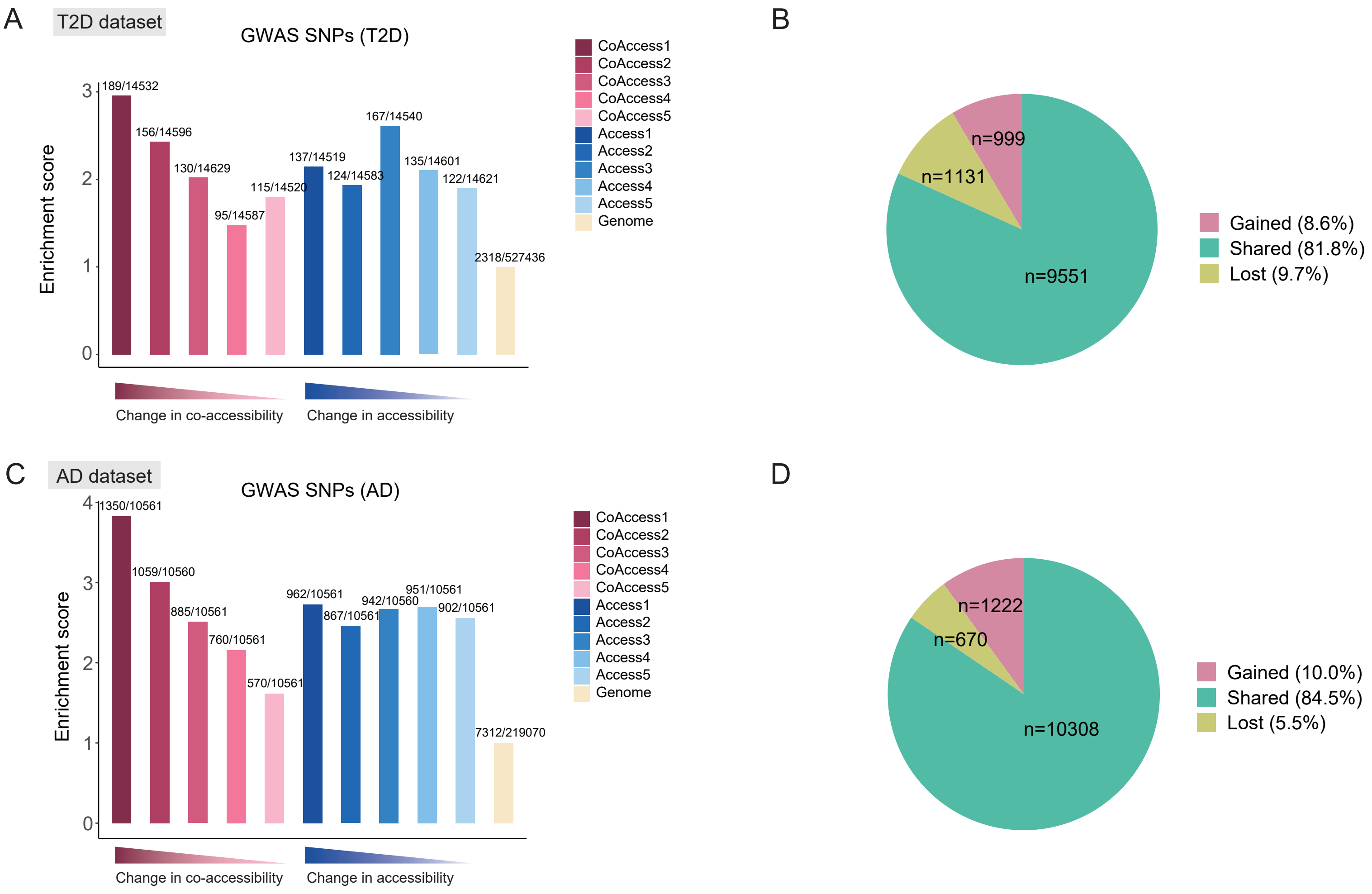

Supplementary Figure S6. Network comparison analysis in T2D and AD datasets. Related to **Figure 6**. (**A** and **C**) Bar plot showing the enrichment of GWAS SNPs in diverse groups ordered by the change of co-accessibility (red) as well as accessibility (blue) in T2D (**A**) and AD (**C**) dataset, respectively. (**B** and **D**) Pie chart displaying the percentages of these three types of enhancer network including Gained, Shared and Lost in T2D (**B**) and AD (**D**) dataset, respectively.

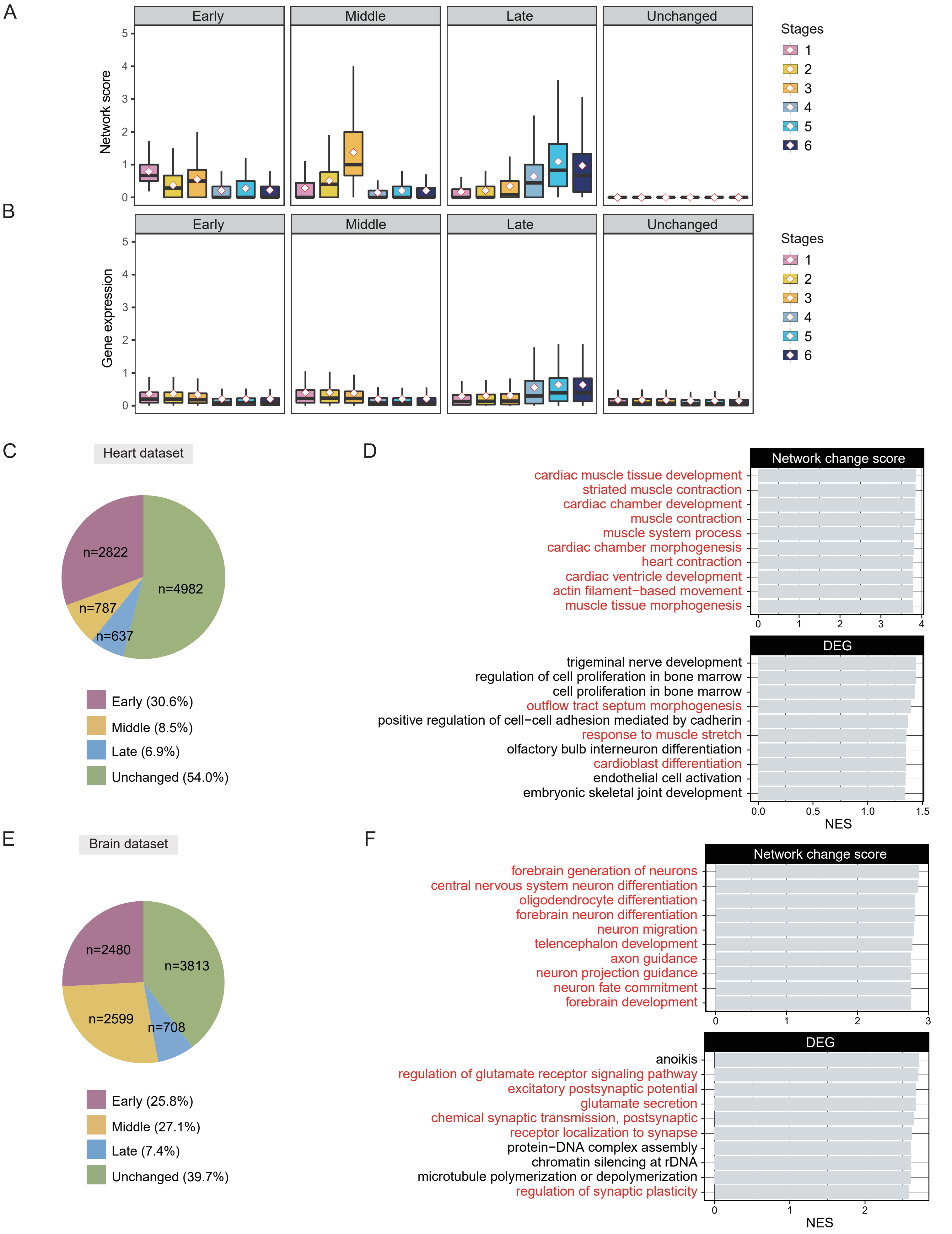

Supplementary Figure S7. Network dynamics analysis in human heart and brain datasets. Related to **Figure 6**. (**A** and **B**) Boxplots displaying the distribution of network score (**A**) and gene expression (**B**) for each group of enhancer networks (including Early, Middle, Late and Unchanged) across different stages during CD8+ T cell differentiation. (**C** and **E**) Pie chart displaying the percentages of these four types of enhancer network including Early, Middle, Late and Unchanged in heart (**C**) and brain (**E**) dataset, respectively. (**D** and **F**) Top 10 significantly enriched BP pathways as determined by GSEA, based on network change score (top) and log2FC value of DEG (bottom), with tissue-related pathways highlighted in red for the heart (**D**) and brain (**F**) datasets, respectively.

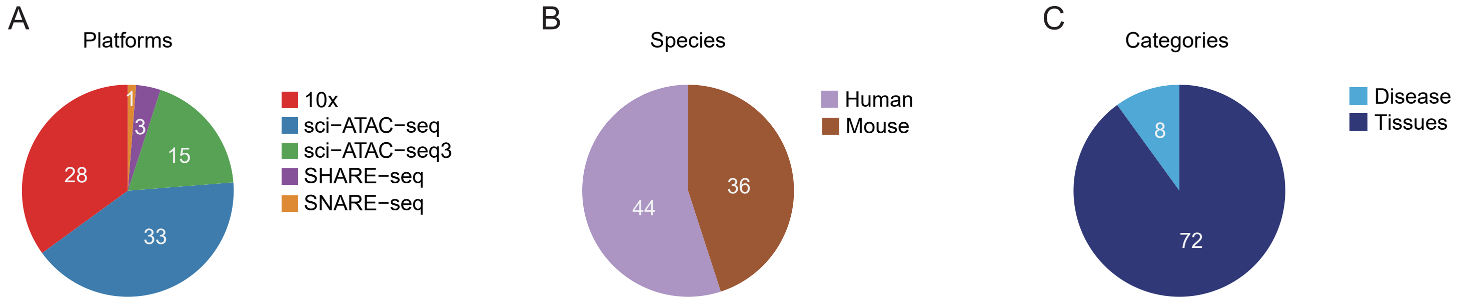

Supplementary Figure S8. Database statistics. Related to **Figure 7**. (**A-C**) The distribution of datasets for different platforms (**A**), species (**B**) and categories (**C**), where "Tissues" represents normal tissues during development and "Disease" represents diseased tissues.
